# Beyond two dimensions: Exploring 3D dielectrophoresis for microparticle control using carbon electrodes

**DOI:** 10.1371/journal.pone.0310978

**Published:** 2024-09-26

**Authors:** Oscar Pilloni, Marc Madou, Laura Oropeza-Ramos

**Affiliations:** 1 Instituto de Ingeniería, Universidad Nacional Autónoma de México, Ciudad de México, México; 2 Instituto Tecnológico y de Estudios Superiores de Monterrey, Monterrey, Nuevo León, México; 3 Facultad de Ingeniería, Universidad Nacional Autónoma de México, Ciudad de México, México; Khalifa University of Science and Technology, UNITED ARAB EMIRATES

## Abstract

This study explores the frontiers of microparticle manipulation by introducing an actuator platform for the three-dimensional positioning of microparticles using dielectrophoresis (DEP), a technique known for its selectivity and ease of integration with microtechnology. Leveraging advancements in carbon-based devices due to their biocompatibility and electrochemical stability, our work extends the application of DEP from two-dimensional constraints to precise 3D positioning within microvolumes, employing a photolithography-based fabrication process known as Carbon-MEMS technology (C-MEMS). We present the design, finite element simulation, fabrication, and testing of this platform, which utilizes a unique combination of planar and 3D carbon microelectrodes individually addressable on a transparent substrate. This setup enables the application of DEP forces, allowing for high-throughput manipulation of multiple microparticles simultaneously, as well as displacement of individual microparticles in any desired direction. Demonstrated with spherical 1μm and 10μm diameter polystyrene microparticles, this platform features straightforward fabrication and is suitable for batch industrial production. The study concludes with a discussion of the platform’s advantages and limitations, marking a significant step toward a valuable tool for studying complex biological systems.

## Introduction

The capacity to study particles with precision at the microscopic level is crucial across various scientific disciplines: from understanding the chemical processes, physiological mechanisms, and physical structures essential for life, to studying diseases and microorganisms. Various methodologies facilitate these studies, such as micromanipulation techniques (e.g. optical tweezers or mechanical micromanipulators), fluorescent microscopy, cytometry, scanning probe microscopy, Raman microspectroscopy, and biological microelectromechanical systems (BioMEMS) [1–5].

In particular, micromanipulation is fundamental for the positioning or isolating of target bioparticles suspended in a liquid medium. Many approaches have been investigated, including optical, thermal, mechanical, magnetic, chemical, and electrical [4, 5]. One of the main strategies developed to achieve micromanipulation uses nonuniform electric fields to polarize the bioparticle and induce a net force on it [6, 7]. This phenomenon is known as dielectrophoresis (DEP), and it is attractive for micromanipulation because it is a rapid, label-free, and bioparticle-safe method that can be easily integrated into microfluidic devices [8].

The theory and principles of DEP were reviewed by B. Çetin et al. [9] and the status of the theory, technological advances, and applications were summarized by R. Pethig [10]. In other reviews, DEP devices have been classified according to the microelectrode array configurations employed to exert the DEP force or by the device’s purpose: trapping, separation, positioning, etc. [4, 11]. Expanding on this latter purpose, it has been shown that dielectrophoresis can be successfully used to position bioparticles in a plane inside a microchannel; nonetheless, it also has the potential to be used for tridimensional positioning of bioparticles inside a microvolume [12, 13]. Additionally, the effect of DEP forces on the viability of bioparticles must be considered, as addressed in [14, 15].

Farasat et al. [11] discussed various microelectrode types, their fabrication, and actuation methods for generating active DEP forces inside microfluidic channels. However, the fabrication of microelectrode geometries is challenging and demands specialized infrastructure, including equipment for metal deposition and controlled environments such as acid fume hoods. Recently, DEP has been implemented in microdevices using the carbon-MEMS (C-MEMS) technology. C-MEMS combines traditional patterning techniques, such as photolithography, with pyrolysis to fabricate micro/nano-glass-like carbon features derived from a patterned organic precursor [16, 17]. Although carbon precursors can be procured from a plethora of polymeric materials, photoresists are commonly used because of their industrial scalability and availability. Also, their use allows for the fabrication of both planar (2D) and volumetric (3D) miniaturized electrodes directly on the final device’s substrate [18–20]. Especially for conventional 3D electrodes, like those based on Au or Pt, this process is typically more costly and complex. This capability allows for the implementation of practically all DEP strategies discussed in [7, 11].

Several reports cover C-MEMS devices that achieve DEP micromanipulation separation techniques such as trapping, sorting, or positioning particles, with the added benefit that carbon microelectrodes exhibit advantageous properties such as electrochemical stability and biocompatibility [16, 21, 22]. Moreover, the biocompatibility of carbon has also been demonstrated. The pyrolyzed carbon material can be used as a substrate for cellular growth [23] and serves as a next-generation material for biomedical devices, such as suitable scaffolds [24] for human neural stem cell growth [25], useful in tissue engineering by providing a 3D framework that supports cell growth and tissue formation, replicating the extracellular matrix for cell attachment and differentiation.

This study proposes a micro platform based on a microelectrode array that combines individually addressable planar and 3D carbon microelectrodes to exert DEP forces to control target microparticles inside a microvolume. This microelectrode array could be used to implement different DEP strategies mentioned above, such as high throughput or single microparticle manipulation. It might be used for tissue engineering, allowing the positioning of cells in specific scaffold locations, and generating virtual scaffolds inside a control volume, or for trapping a single bioparticle for a biomedical study. Furthermore, the fabrication technology supports the use of transparent substrates, which are advantageous for monitoring the produced devices with conventional microscopes. It is also well-suited for batch production and offers all the benefits associated with carbonaceous micro-structured materials.

The design performance of our device was simulated using finite element analysis software (COMSOL) to verify its viability before fabrication. Subsequently, the proposed design was fabricated on a transparent substrate using C-MEMS technology [18]. Finally, a proof of concept of the micro platform was performed using classical DEP to position plastic fluorescent microparticles inside a microvolume, allowing us to compare the actual performance against the anticipated outcomes derived from the simulation results. A detailed description of each step in this process is provided in the sections that follow. The experimental parameters and conditions were selected to minimize other AC electrokinetic motion effects such as electrothermal forces (ETF), AC electroosmosis (ACEO), and electrokinetic flow phenomena that could be coupled to DEP under other conditions, as discussed in depth in [26, 27]. Finally, the advantages and limitations of the proposed platform are discussed.

## Design considerations

### Principles of individually addressable carbon electrodes for 3D DEP positioning

The foundational concept behind the suggested design is rooted in the dielectrophoretic (DEP) force, ${\vec{F}}_{DEP}$, imparted on a particle through polarization. The DEP force can be calculated based on the multipole moment ($\vec{p}$) that arises from the overall charge density and the intensity of the electric field ($\vec{E}$), as shown in Eq 1.


$${\vec{F}}_{DEP}=\vec{p}∙\nabla \vec{E}$$ (1)

The polarization-induced moment in a dielectric particle exposed to an external electric field is derived from analyzing the induced multipole moments within a spherical particle, where the dipole moment is a particular example. By assessing these moments, one can ascertain the dielectrophoretic (DEP) force. This approach considers the effects of polarization and the resultant effective dipole moment, which emerges from the charge distribution in response to the external electric field.

Starting off with a fundamental model that assumes a homogeneous and isotropic sphere, defined by its radius (*R*_*p*_) and complex electrical permittivity (*ϵ*_*p*_), submerged in a medium with a distinct permittivity (*ϵ*_*m*_), this method models the sphere’s polarization and the resulting electric potential. Key to this model are boundary conditions that guarantee the electric potential’s continuity across the interface between the particle and the surrounding medium, the uniformity of the electric potential gradient’s normal component on the particle’s surface, and the fulfillment of Laplace’s equation across the entire volume. T. B. Jones et al extended this research to derive the nth multipole moment of a particle influenced by a dielectrophoretic effect (Eq 2) [28]. Note that the Clausius-Mossotti factor (*K*^(*n*)^) has also been redefined to accommodate the nth multipole, which describes the polarizability of a particle in a medium by comparing their complex electrical permittivities ($\epsilon_{p}^{*},\;\epsilon_{m}^{*}$ respectively), as shown in Eq 3. The complex electrical permittivity (ϵ*) is defined by the electrical permittivity (*ϵ*), the conductivity of the material (*σ*) and the angular frequency of the electric field (*ω*) as shown in Eq 4.


$${\vec{p}}_{n}=\frac{4\pi n\epsilon_{m}}{(2n-1)‼}K^{\left(n\right)}R_{p}^{2n+1}{\left(\nabla \right)}^{\left(n-1\right)}\vec{E}$$ (2)

$$K^{\left(n\right)}=\frac{\epsilon_{p}^{*}-\epsilon_{m}^{*}}{n\epsilon_{p}^{*}+(n+1)\epsilon_{m}^{*}}$$ (3)

$$\epsilon^{*}=\epsilon -i\left(\frac{\sigma }{\omega }\right)$$ (4)

Utilizing the ${\vec{p}}_{n}$ multipole and organizing terms via dyadic notation, the comprehensive formula for the dielectrophoretic force associated with the nth multipole moment can be derived, as outlined in Eq 5. Here, the notation [∙]^*n*^ signifies the nth iteration of the dot product applied to the dyadic tensor, while (*∇*)^*n*^ indicates the application of n gradient operators to the electric field. Only the real part of the expression is used to obtain the DEP force.


$${\vec{F}}_{DEP_{n}}=Re\left[\frac{{\vec{p}}_{n}}{n!}{\left[∙\right]}^{n}{\left(\nabla \right)}^{n}\vec{E}\right]$$ (5)

By elaborating on Eq 5 to compute the time-averaged dielectrophoretic (DEP) force and addressing the scenario of a dielectric and spherical particle suspended in a medium subjected to a non-uniform electric field, we derive Eq 6.


$$<{\vec{F}}_{DEP}>=\frac{1}{2}\sum_{n=1}^{\infty }Re\left[\frac{4\pi \epsilon_{m}K^{\left(n\right)}R_{p}^{2n+1}}{(n-1)!(2n-1)‼}{\left(\nabla \right)}^{\left(n-1\right)}\vec{E}{\left[∙\right]}^{n}{\left(\nabla \right)}^{n}\vec{E}\right]$$ (6)

Typically, Eq 6 is streamlined to calculate the time-averaged DEP force corresponding to the dipole moment (*n* = 2) in a spherical microparticle, as specified in Eq 7. Higher degree multipoles are usually disregarded due to their diminishing contribution to the overall force. This simplification also facilitates the computation of the Clausius-Mossotti factor (*Re*[*K*]), leading to Eq 8 [10].


$$<{\vec{F}}_{DEP}>=2\;\pi \;\epsilon_{m}Re[K]R_{p}^{3}\nabla {\vec{E}}^{2}$$ (7)

$$K=\frac{\epsilon_{p}^{*}-\epsilon_{m}^{*}}{\epsilon_{p}^{*}+\epsilon_{m}^{*}}$$ (8)

**Fig 1:**
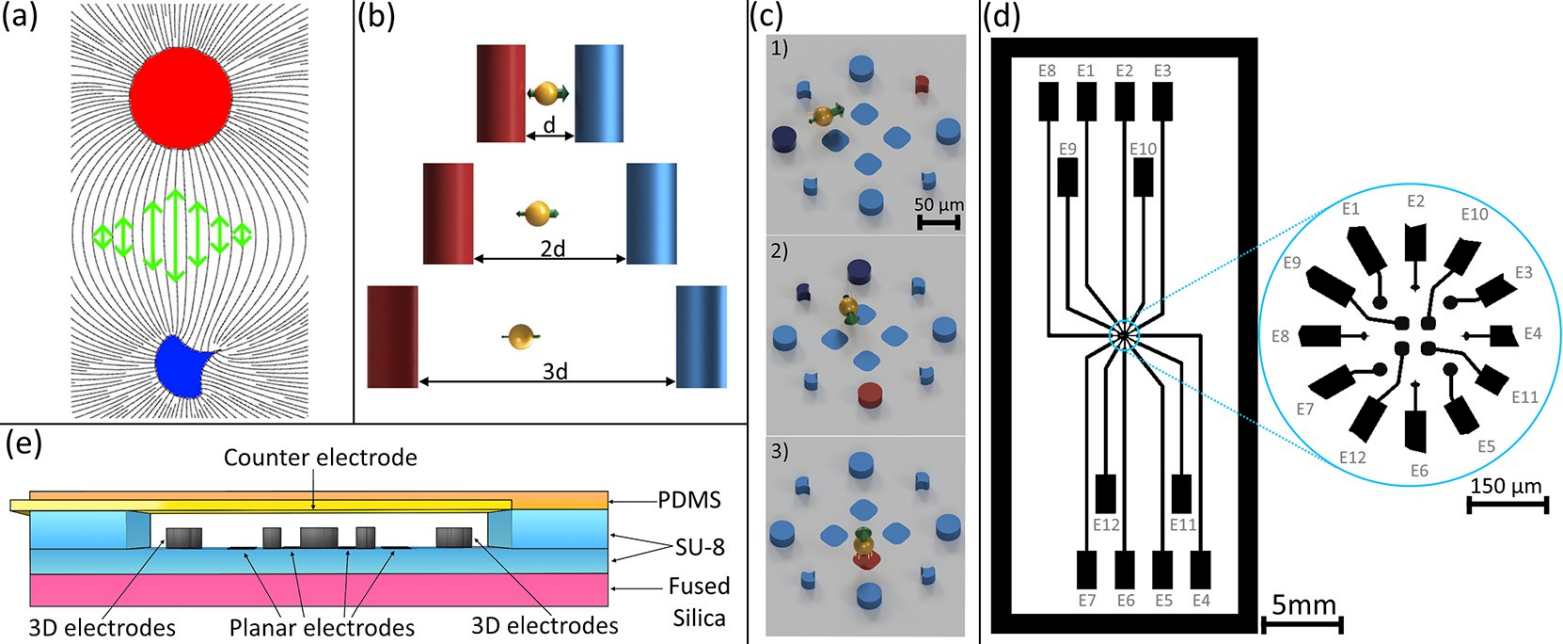
Design overview of the proposed system. The conceptual framework involves (a) generating an electric field by placing two electrodes in opposition—one connected to an alternating current (AC) voltage source (*V*_*DEP*_, depicted in red) and the other grounded (*GND*, shown in blue). The visualized lines represent the trajectory along which particles move under the influence of dielectrophoresis (DEP, green arrows), with the actual path varying based on the DEP force being either positive or negative. (b) The DEP force strengthens with closer electrode spacing, creating a trade-off between force magnitude and control volume size to manipulate a microparticle (yellow sphere). (c) The concept design of the microelectrode array was developed based on the insights gained from (a) and (b), allowing for three-dimensional microparticle manipulation. (d) The design incorporates selectable pairs of three-dimensional (3D) electrodes to direct particle movement along the X and Y axes, and planar electrodes paired with a top counter electrode, enabling vertical (Z-axis) particle movement, shown in the cross section of the device (e).

These equations reveal that the time-averaged trajectory of a particle influenced by dielectrophoretic force aligns with the electric field’s gradient. Essentially, the path of the DEP force vector parallels a line that links the pair of electrodes, generating a nonuniform electric field, as illustrated in Fig 1A. Moreover, the DEP force is inversely proportional to the distance between the exerting electrodes, being stronger when the electrodes are closer together. This presents a conundrum: to manipulate particles within a large volume, the electrodes must be widely spaced, as they define the control volume. However, spacing the electrodes too far apart weakens the DEP force. Conversely, placing the electrodes closer together enhances the magnitude of the DEP force but reduces the size of the control volume, as illustrated in Fig 1B.

These conflicting requirements informed the development of an optimized microelectrode array that effectively balances both demands to manipulate particle positioning within a microvolume. By employing individually controllable electrodes arranged around a designated volume, specific electrode pairs can be activated to create an electric field that applies a DEP force to a single or large number of particles, guiding them along the lines connecting the electrodes. Three-dimensional manipulation is achieved through strategic electrode placement, as depicted in Fig 1C and 1D, combining planar and 3D electrodes to simplify fabrication via multilayer photolithography and pyrolysis. In order to demonstrate the platform experimental capabilities, we choose planar electrodes paired with a counter electrode to drive a particle motion along the Z-axis (Fig 1E), while 3D electrodes are employed to control motion along the X and Y axes, as is also detailed in the simulation section. Please note that although these configurations achieve motion along lines that connect the chosen electrodes, multiple combinations of these electrodes could be used to induce other paths of motion. Ultimately, the direction of particle movement within the chosen medium is dictated by their polarizability: particles move towards electrodes with a positive real part of the Clausius-Mossotti factor (Re[K]) and away from those with a negative value. This flexibility enables control of particle positioning within the defined microvolume.

The proposed microelectrode array was designed to exert a DEP force within a microvolume of approximately 100^3^ μ*m*^3^. This volume was chosen to accommodate the size of common bioparticles (such as mammalian cells, ~10 μm in diameter). The shape of the electrode array is selected to encase this volume. The limiting factor of the volume size is the voltage required to induce the DEP force in the particles. Higher voltages are required to establish an electric field that is adequate to effectively apply the DEP force in larger volumes.

Four rounded squares (30 μm sides) were designed for the planar electrodes, and four cylinders (30 μm diameter) and four spikes (20 μm length) were designed for the 3D electrodes. These lengths were selected to ensure electrode sizes were comparable to mammalian cells and to accommodate the constraints imposed by our microfabrication facilities. Schematics for the microelectrode array and accompanying microfluidics are provided in S1 Appendix.

**Fig 2:**
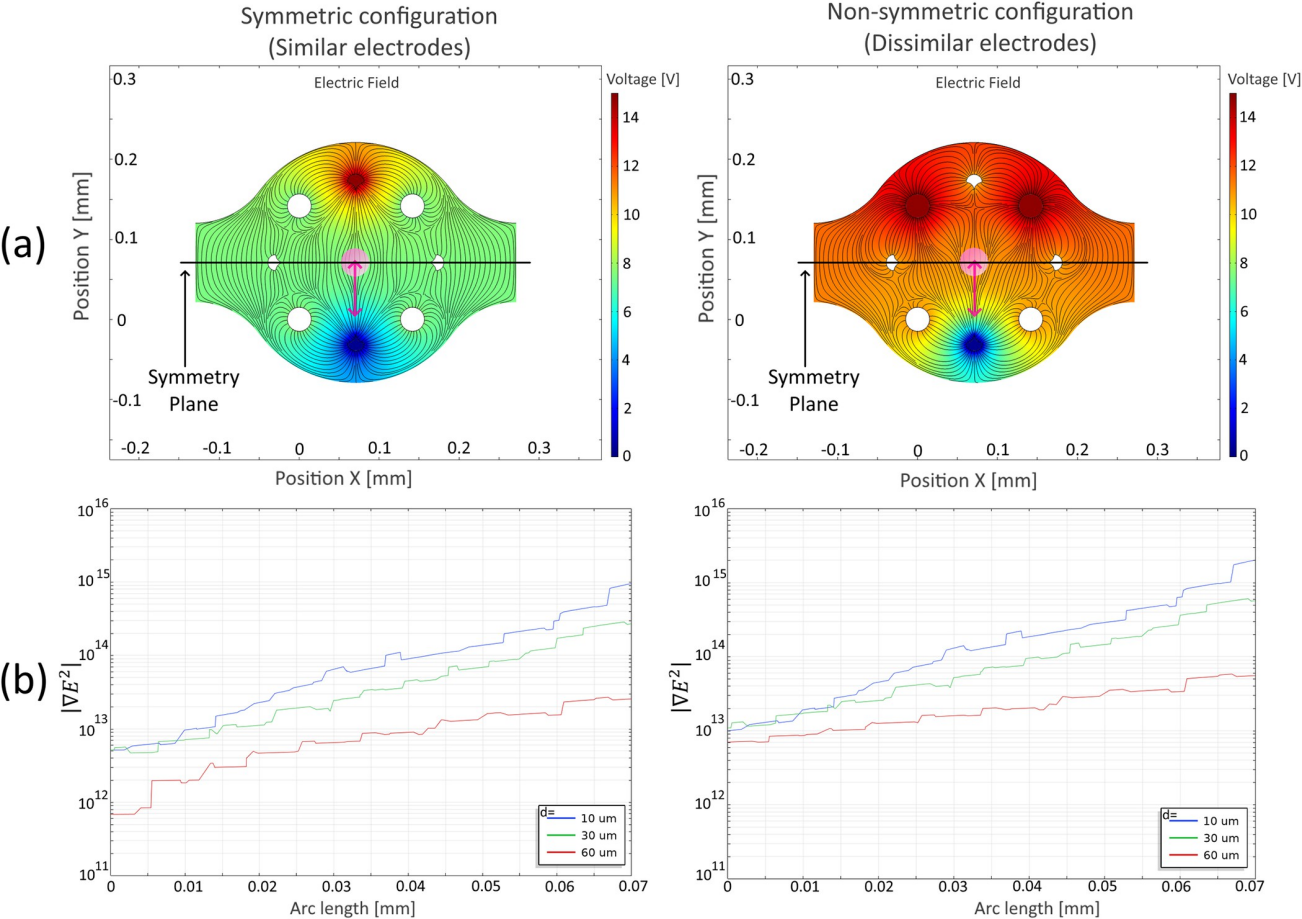
Effects of symmetry on two example array configurations. These configurations were selected to achieve movement of a target particle (pink circle) along the intended pink line of motion. Symmetry is considered regarding a plane that divides the microelectrode array by the middle and is perpendicular to the intended line of motion. The left configuration is symmetric to the marked plane and uses similarly shaped electrodes. The right configuration is non-symmetric to the marked plane and uses different electrode shapes on either side of the symmetry plane. (a) Shows the electric field established for either electrode configuration inside the microchannel. (b) Shows the magnitude of *∇E*^2^ for the length of the pink line of motion. *F*_*DEP*_ is directly proportional to the magnitude of *∇E*^2^ (Eq 1). Note that |*∇E*^2^| is roughly ten times stronger in the non-symmetric configuration. It is due to this fact that we decided to use different shapes for the electrodes that integrate the array. Red electrodes have applied *V*_*DEP*_ = 15 V, sine wave, 100kHz. Blue electrodes are tied to electrical ground.

It is important to note that this design allows for multiple microelectrode configurations where identical electrode shapes can be employed to generate the electric field. Nonetheless, variations in electrode material and surface imperfections resulting from fabrication can influence the distribution of the electric field. Additionally, the spatial arrangement and distance between electrodes can create zones with high gradients in the electric field, as depicted in Fig 2 on the left side. However, employing non-symmetrical electrode configurations is preferred as it enhances the electric field gradient. By combining dissimilar electrode shapes with non-symmetrical electrode positioning, it becomes feasible to augment the necessary non-uniformity of the electric field for effective dielectrophoresis, as illustrated in Fig 2 on the right side. This strategy proves particularly effective when aiming to avoid a symmetric array configuration relative to a symmetry plane dividing the control volume centrally and perpendicular to the intended direction of particle motion.

Finally, to accurately determine the position and trajectories of microparticles influenced by DEP, it is essential to consider the Navier-Stokes equation, Eq 9. In this context, *ρ* is the fluid density, $\bar{u}\;\bar{u}$ is the velocity dyadic tensor, $\hat{n}$ is the surface-normal unit vector and *τ* is the fluid stress tensor. The term $\bar{f_{i}}$ involves other external forces applied to the particle, such as the DEP force, the drag force and the buoyancy force.


$$\frac{\partial }{\partial t}\int_{V}\rho \;\bar{u}\;dV=-\int_{S}(\rho \;\bar{u}\;\bar{u})∙\hat{n}\;dA+\int_{S}\bar{\bar{\tau }}∙\hat{n}\;dA+\int_{V}\sum_{i}\bar{f_{i}}\;dV$$ (9)

Eq 9 can be simplified to the Stokes flow equation (Eq 10) by neglecting the inertial terms, owing to the small characteristic lengths of the microdevice. This simplification is valid because, at such scales, the Reynolds number is very low, rendering the inertial forces negligible compared to the viscous forces. Here, *P* is the pressure field, *μ* is the dynamic viscosity of the fluid and $\bar{u}$ is the velocity field.


$$\nabla P-\mu \nabla^{2}\bar{u}=\sum_{i}\bar{f_{i}}$$ (10)

### Simulation of the electric field for the microelectrode design

The proposed microelectrode array was modeled using CAD software (Autodesk Inventor) and simulated using finite element simulation (COMSOL) to obtain the electric field gradient direction and intensity at different positions between the exerting microelectrodes. This information, in conjunction with the computed K factor, allows for the prediction of the particle motion inside the microvolume.

The following parameters and conditions were selected for the simulations and the experiments described in the following section:

- A sine wave with an amplitude of 15 V_pp_ and a frequency of 100 kHz was used as the signal source (*V*_*DEP*_) to induce electrokinetic phenomena.
- The *V*_*DEP*_ signal was toggled on and off to produce a controlled and momentary application of DEP force (enabling *V*_*DEP*_ for 10 s).
- Deionized and bi-distilled water was used as the aqueous medium.
- Fluorescent microspheres (Thermo Fisher, nylon G1000, 10 μm diameter) were used as the test particles. Their size was selected to simulate average mammalian cells.

The electrical properties of these elements are summarized in Table 1. The combination of these electrical parameters has been found to produce strong DEP forces [29] while concurrently yielding minimal or negligible ACEO flow [30]. Table 2 provides an overview of additional finite element simulation parameters.

**Table 1:** Suspension media and microparticle properties used in the finite element analysis simulations.

Properties	Value
**Suspension media**	**Deionized and distilled water**
Electrical conductivity	$\sigma_{m}=5x10^{-6}\frac{S}{m}$
Relative electrical permittivity	*ϵ*_*m*_ = 80
Dynamic viscosity	*μ* = 8.9*x*10^−4^ *Pa s*
**Microparticles**	**FluoSpheres (Polystyrene)**
Density	$\rho =1040\frac{kg}{m^{3}}$
Diameter	*ϕ* = 10 *μm*
Electrical conductivity	$\sigma_{p}=6.7x10^{-14}\frac{S}{m}$
Relative electrical permittivity	*ϵ*_*p*_ = 2.6

**Table 2:** Finite element simulation setup parameters.

Parameter	Value
Mesh type	Free tetrahedral
Maximum element size	0.005 [mm]
Minimum element size	0.001 [mm]
Maximum element grow rate	1.5
Number of elements	918341
Study type	Stationary solver
Solver type	Linear solver (Conjugate gradients)
Number of degrees of freedom solved for	1255602
Relative tolerance	0.001
Relative error	5.6*x*10^−5^
Relative residual	5.1*x*10^−7^

**Fig 3:**
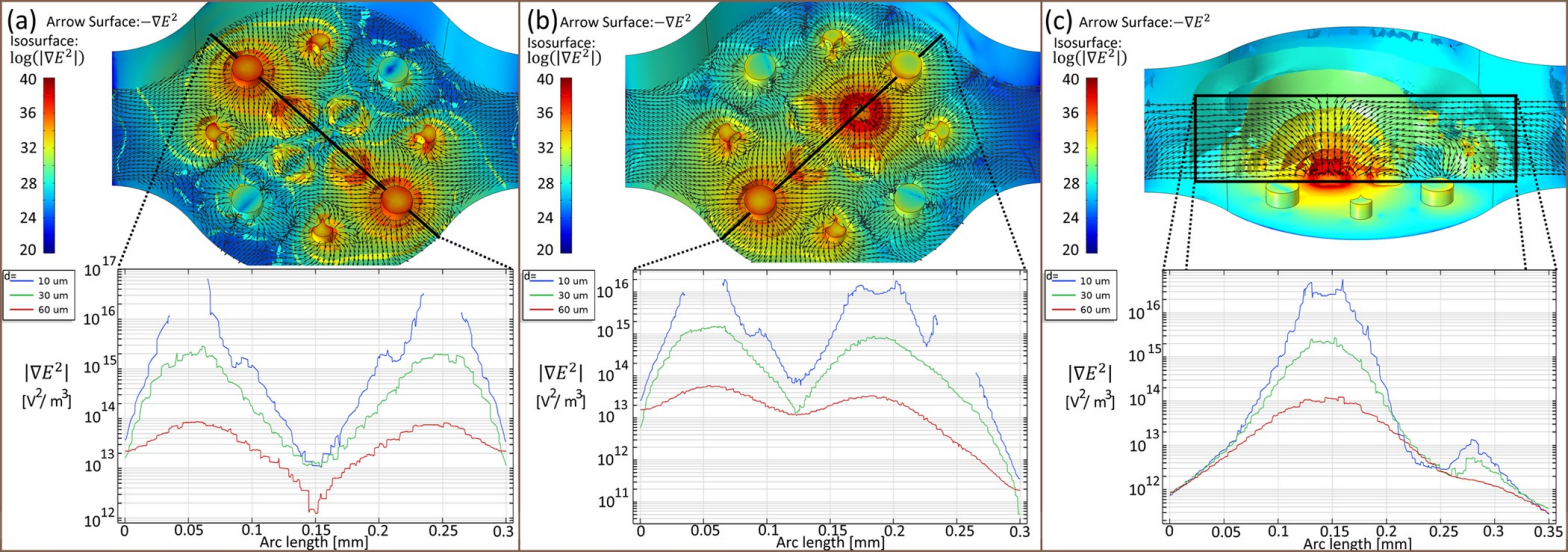
Finite element simulations (COMSOL) of sample microplatform configurations. (a) and (b) configurations were selected for micromanipulation in the XY plane, while (c) was selected for the Z direction. Specific electrode array configurations and applied voltages are described in S2 Appendix.

Finite element analysis was conducted on the proposed microelectrode array design to ascertain the direction and squared magnitude of the electric field gradient between various test configurations of the selected electrode pairs, as outlined in Table 3. Fig 3 illustrates the direction and intensity of the electric field gradient at various positions between the applied microelectrode configurations. These configurations were specifically chosen as illustrative examples for testing DEP micromanipulation across all three axes of motion.

**Table 3:** Test array configurations are graphically shown in the finite element simulations of Fig 3.

Test Configuration	Intended direction of particle movement	*V*_*DEP*_ Electrode	*GND* Electrode
(a)	XY plane	3D cylinder	3D cylinder
(b)	XY plane	3D cylinder	Planar square
(c)	Z direction	Planar square	Top counter electrode

Specific electrodes used for each configuration are detailed in S2 Appendix.

In Fig 3, the top images illustrate the logarithm of the gradient of the squared electric field as a series of isometric surfaces within the control volume, representing the strength of the DEP force (where red denotes stronger force). Following the dipole moment model of dielectrophoresis force, arrows depict the electric field vectors at specific planes within the volume for each electrode array configuration. These planes correspond to the anticipated trajectories of particle motion (trajectories) for each array configuration, with their positions chosen arbitrarily as an example of potential particle trajectories based on their initial positions within that plane. The arrow direction indicates the direction of particle motion in these planes considering negative DEP force. Fig 3A and 3B display this information for a XY plane with Z = 10 μm, while Fig 3C presents a XZ plane with Y = 45 μm.

The lower graphs of Fig 3 depict the magnitude of the squared electric field at various "d" heights measured orthogonally from the planar electrodes and along the black solid line within the control volume. This magnitude is plotted for d = 10, 30, and 60 μm as arbitrarily selected sample heights. The maximum gradient of the electric field is observed near the test electrodes, indicating that the maximum DEP force is applied at these positions, while the minimum gradient is typically found around the midpoint of the selected pair of electrodes. Additionally, the arrow surfaces demonstrate that the chosen configurations predominantly induce DEP force movement along the intended directions.

The simulations reveal distinct patterns in the expected particle motions. Configurations (a) and (b) are predicted to predominantly induce particle movement within the XY plane, while configuration (c) is anticipated to direct motion along the Z-axis. Simulation results demonstrate that the maximum DEP force occurs in close proximity to the selected electrodes, with the force magnitude directly proportional to the magnitude of ${\vec{E}}^{2}$ (Eq 6). Consequently, particles situated closer to the electrodes experience a stronger DEP force. Conversely, the force magnitude diminishes with increasing particle height (d) within the control volume. This suggests that particles suspended at higher positions within the control volume would exhibit greater resistance to movement. Additionally, arrow surfaces were plotted to depict the negative DEP force for each configuration tested, offering directional guidance for particle motion in the proof-of-concept experiments discussed in the following sections.

## Materials and methods

### Fabrication of carbon microelectrodes on a transparent fused silica substrate

**Fig 4:**
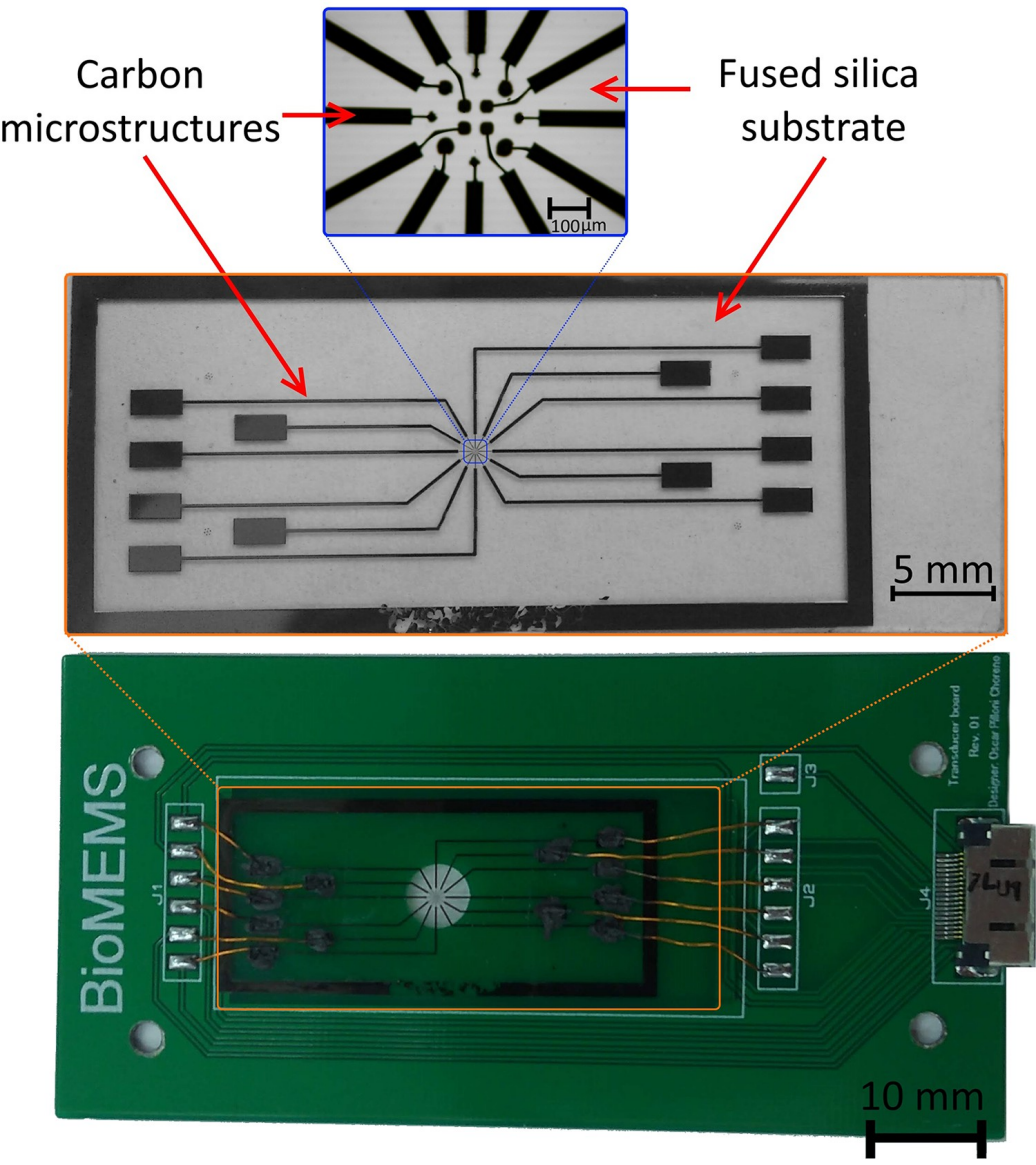
Complete microelectrode C-MEMS device post-pyrolysis. (Top) Micrograph depicting the fabricated microelectrode array. (Middle) Full layer stack featuring the passivation layer. (Bottom) Device affixed onto a custom PCB for mechanical support and electrical connections.

The microelectrode array was fabricated using a combination of photolithography and pyrolysis, as described in a previous work [18, 31]. S3 Appendix contains the fabrication methodology and parameters. In brief, SU-8 3035 was used to photolithographically pattern five photoresist layers that conform to the microelectrode array and microchamber. Pyrolysis was performed on the first three layers to obtain carbon features, which formed electrically conducting traces, interface pads, and microelectrode arrays. The final pyrolysis temperature was 900°C for an hour. Layer four was then deposited to electrically passivate the areas other than the electrode array and interface pads. Finally, layer five is used to form a microchamber with the microelectrode array at its core. Following fabrication, the carbon MEMS array was affixed onto a customized PCB for electrical connections, as illustrated in Fig 4.

**Fig 5:**
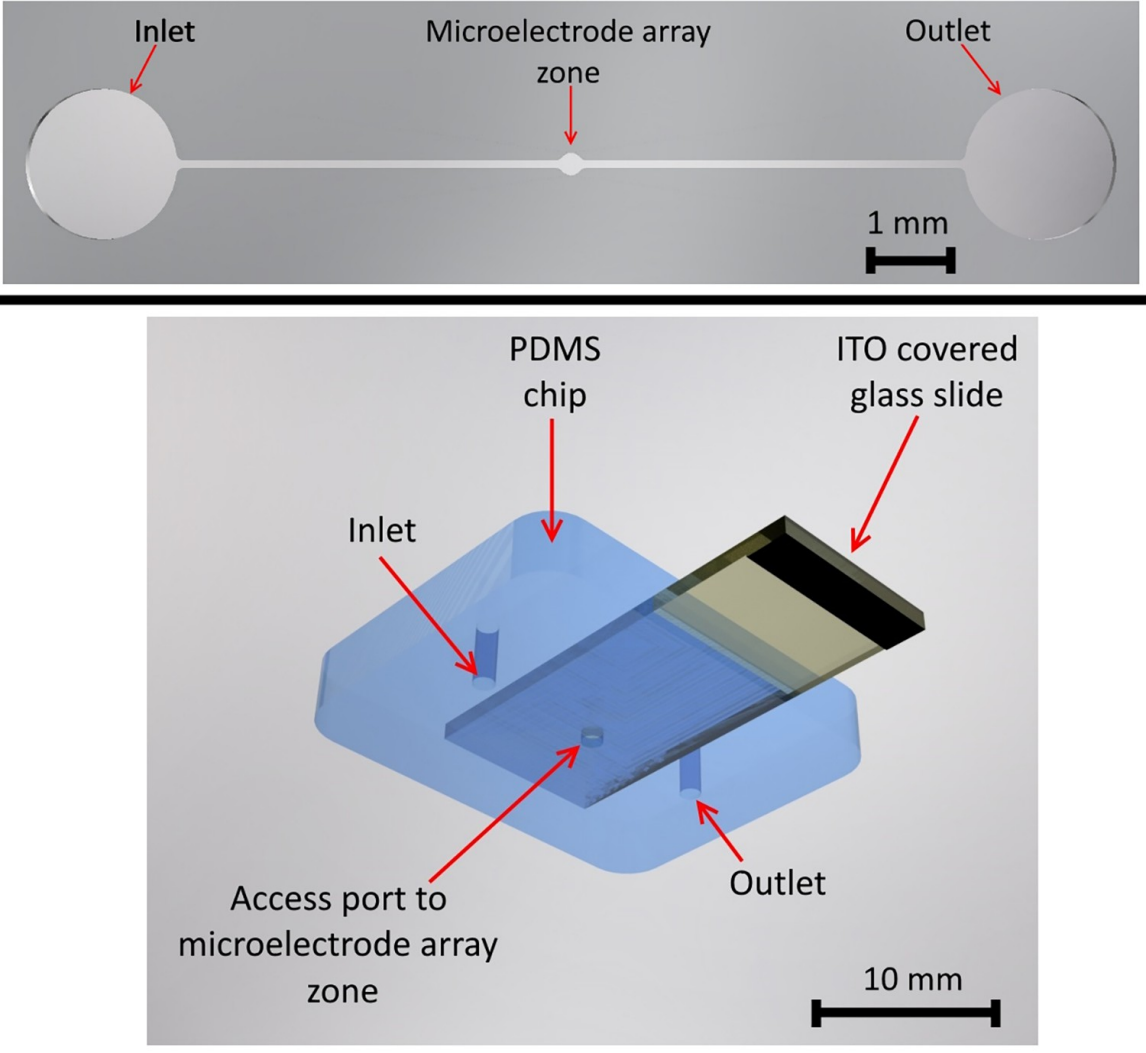
Microfluidic features of the microdevice. (Top) Microchannel layer (Layer 5) built in the microelectrode array module, and (bottom) PDMS chip that adds the counter electrode (ITO covered glass slide) for micromanipulation in the z-axis.

To seal the microfluidic device, a simple chip was fabricated using soft lithography of polydimethylsiloxane (PDMS). This approach enables the incorporation of a glass slide coated with a thin layer of indium tin oxide (ITO), which serves as a counter electrode for the 2D microelectrodes. This setup facilitates z-axis micromanipulation while maintaining optical transparency for observing the microelectrode zone. The PDMS chip is shown in Fig 5.

### Microplatform assembly and setup description

**Fig 6:**
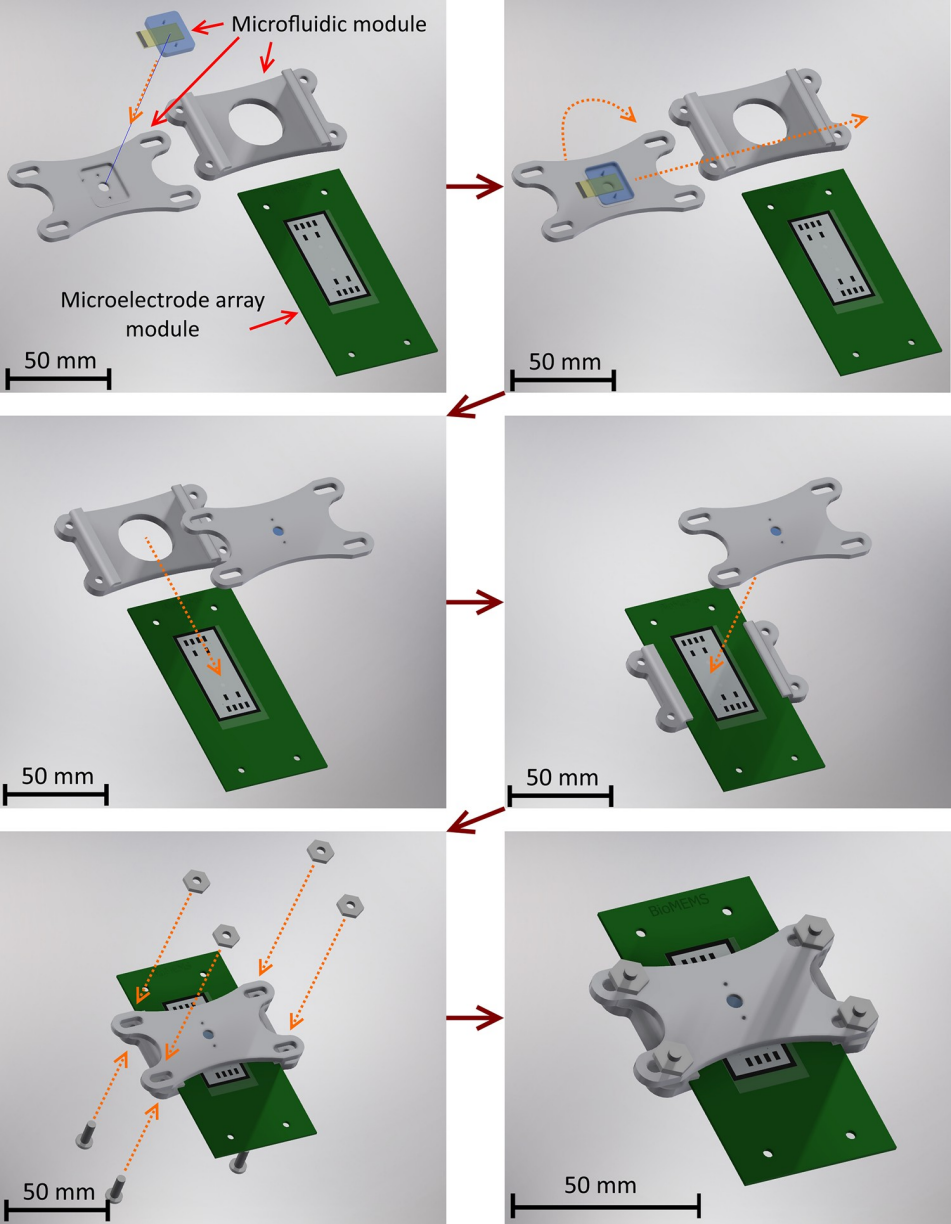
Assembly process of the microplatform. The assembly involved securing both the PCB with the microelectrode array and the PDMS chip using a 3D-printed bracket. This ensured mechanical stability and precise alignment, effectively sealing the microfluidic channel during experiments. Reprinted from [31] under a CC BY license, with permission from Universidad Nacional Autónoma de México, original copyright 2019.

The microplatform was constructed through the integration of the PCB, microelectrode array, and PDMS chip. The assembly process and resulting configuration are elucidated in Fig 6. Initially, the microfluidic module is assembled by affixing the PDMS chip to a 3D-printed mechanical harness, securing and aligning it. Subsequently, the microfluidic module is flipped to its final orientation, and the mechanical harness base is smoothly inserted into the C-MEMS chip with PCB. Aligning the components, the microfluidic module is pressed against the C-MEMS chip, and alignment is fine-tuned using the four bolts securing the entire fixture.

**Fig 7:**
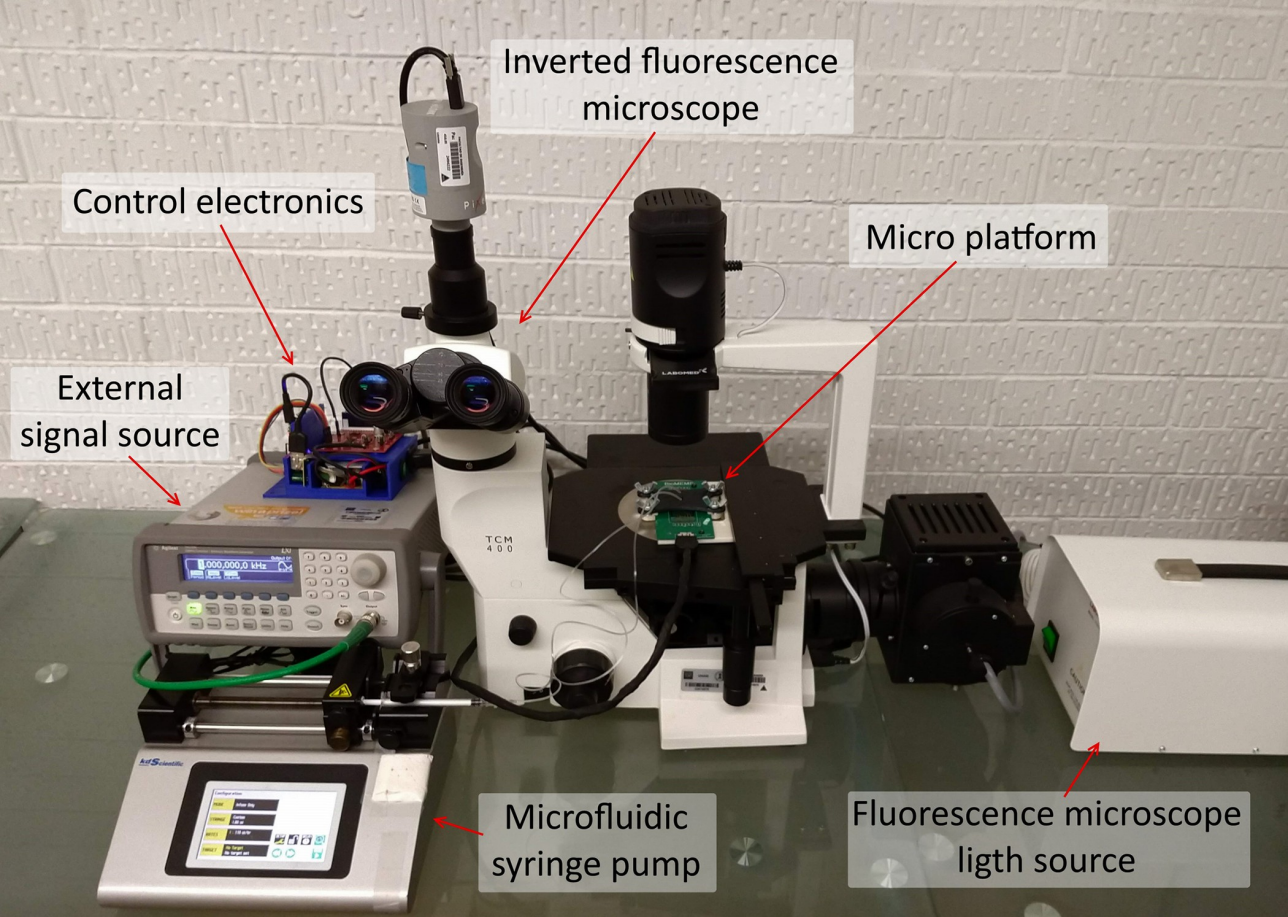
Experimental setup. Photograph of the experimental configuration implemented for conducting the micromanipulation experiments using the C-MEMS micro platform.

Subsequently, the C-MEMS platform was affixed onto a TCM 400 fluorescence inverted microscope for observation and video capture. The PCB was connected to a custom control electronics module. To evaluate the platform’s functionality, a suspension of fluorescent microspheres (Thermo Fisher, nylon G1000, 10 μm diameter) in deionized and bi-distilled water was prepared and loaded into a syringe. This syringe was then placed in a syringe pump and connected to the inlet of the PDMS microfluidic chip. The syringe pump introduced the microspheres suspension into the microchannel, saturating both the channel and the microelectrode array zone. The microarray zone was monitored with the microscope during microparticle loading, and the pump was stopped once the desired number of particles were within the microelectrode array control volume. Using this loading method, the initial positions of the particles within the control volume cannot be precisely controlled and are largely random. However, by repeatedly allowing the suspension to flow and then halting it, the particles were positioned in approximate starting locations corresponding to those in the simulations. After particle loading, a 5-minute dwell time was used to ensure system stabilization, allowing the microspheres to settle and remain stationary within the control microvolume. No external flow rate was applied during experiments to isolate the effects of dielectrophoresis. The complete experimental setup is illustrated in Fig 7.

For each experiment, pairs of electrodes were selected to exert a DEP force (15 V, sine wave, 100 kHz) on a selected particle or group of particles, aiming to move them in the desired direction. Using these parameters, a negative DEP was expected for the particle-media combination (K ~-0.5, calculated using Eq 2). All experiments were conducted at room temperature (~295 K).

## Results and discussion

### Three-dimensional manipulation of multiple particles

**Fig 8:**
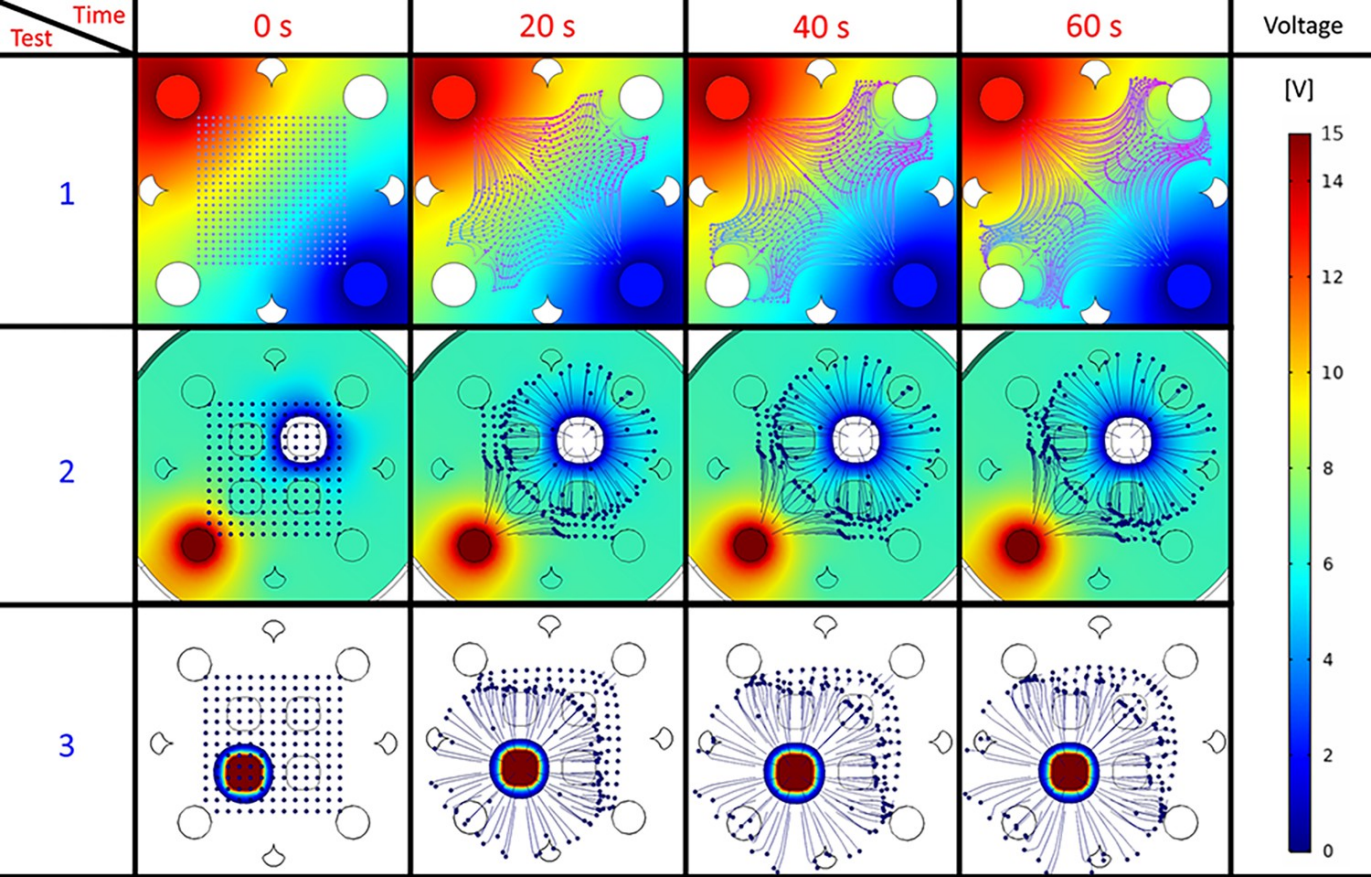
Snapshots of multi-particle micromanipulation simulations. Simulations one and two were designed to primarily generate DEP forces along the observed plane, whereas simulation three was devised to produce trajectories along the axis orthogonal to the view. These configurations were intended to investigate and compare the directional control of particle motion within the microfluidic system.

First, simulations were conducted using a set of multiple particles, simulating their trajectories as directed forces were applied. Particle-to-particle interaction was not simulated. Fig 8 depicts the trajectories developed within the control volume, observed from a top-down perspective. It is important to note that the final positions of the particles result from a combination of the electrodes used for the dielectrophoresis effect and the initial position of the particle in question. This leads the particles to reach an equilibrium point where the dielectrophoresis force no longer exceeds the drag forces within the fluid.

**Fig 9:**
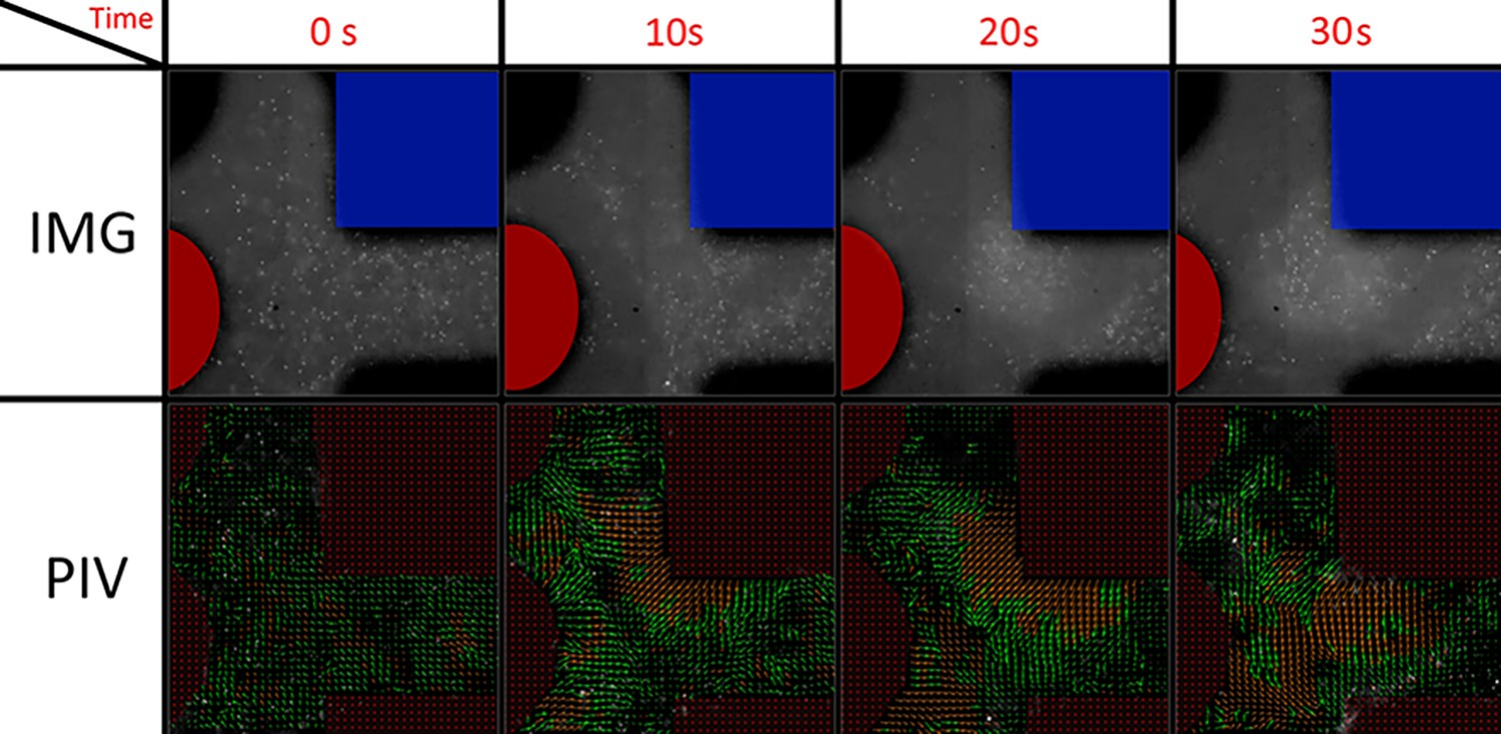
Snapshots of the multi-particle experimental micromanipulation tests and Particle Image Velocimetry (PIV). The electrodes, represented by the black areas, were masked and excluded from the PIV analysis (indicated in red in the lower images). Fluorescent particles captured in the top images underwent processing to derive the velocity vectors shown in the images below. In the initial PIV image, the vectors indicate a slight drift of microparticles. However, as the DEP force was exerted, subsequent vectors illustrate particles moving away from the selected electrodes (visible in the top images, marked in red and blue), a phenomenon consistent with negative dielectrophoresis. This observation corroborates the anticipated particle behavior under the influence of the applied electric field.

Using a similar electrode configuration and experimental parameters as those employed in the simulation shown in Fig 8 test 2, an experiment was conducted using fluorescent microparticles, as depicted in Fig 9 where image frames were processed to obtain Particle Image Velocimetry (PIV) data at different intervals throughout the experiment. This technique enables the measurement of particle velocities within the microfluidic system, providing insights into the fluid dynamics and particle behavior under the influence of the applied electric field. It is observed that once the electric field is established, the dielectrophoresis effect generates a constant particle movement consistent with negative dielectrophoresis, wherein particles move away from the outer electrodes and tend to form a line around the center of them. This observation aligns with the simulation results for the expected microparticle manipulation behavior.

### Single particle micromanipulation

**Fig 10:**
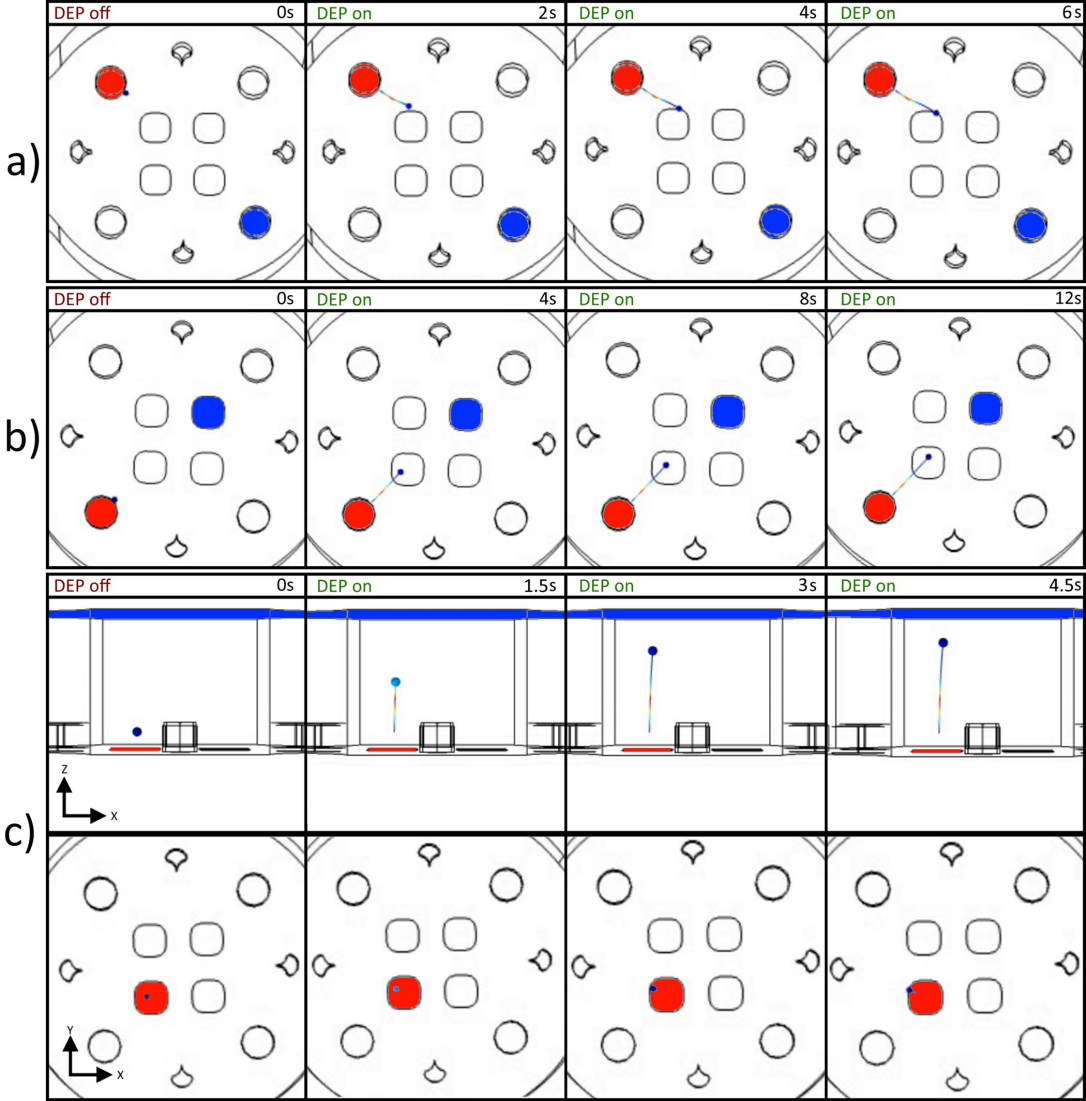
Snapshots of individual particle micromanipulation simulations. Negative DEP force was consistently observed across all simulations. The images illustrate active electrodes generating electric potential (highlighted in red) and ground electrodes (highlighted in blue), which induce a DEP force on the target microparticles. Upon activation of the DEP force, configurations (a) and (b) exerted particle motions mostly along the XY plane, whereas configuration (c) demonstrated motion along the Z-axis. Additionally, panel (c) provides both top-to-bottom and side views for clarity.

**Fig 11:**
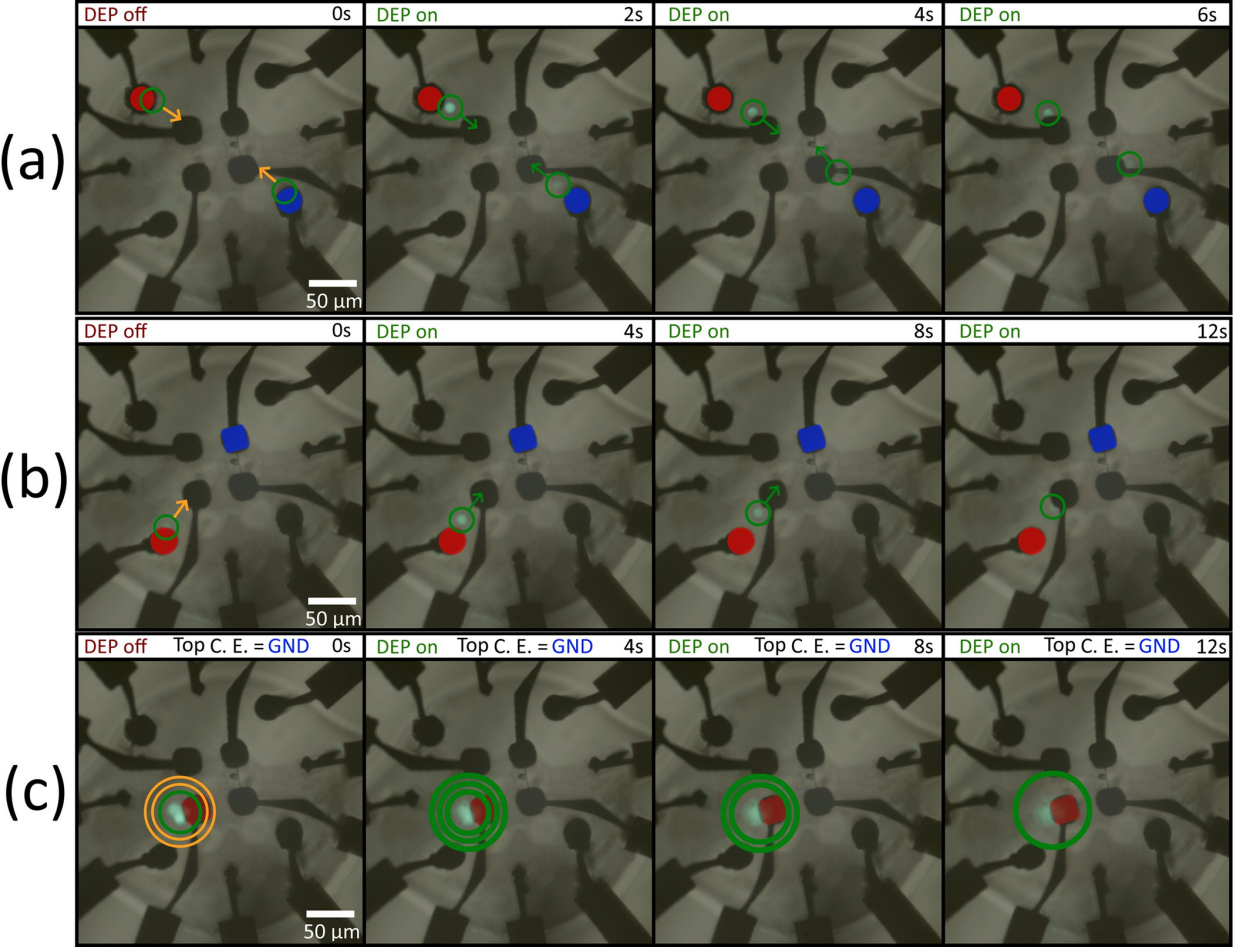
Snapshots of single particle experimental 3D micromanipulation tests. Fluorescent nylon microspheres were used as sample microparticles. A negative DEP force was observed in all tests. Images show active electric potential electrodes (red) and ground electrodes (blue) that induce a DEP force on the target microparticles (green circles). The intended direction of motion for each configuration is shown in the first frames (orange indicators). Once the DEP force was turned on, the particles moved in the direction shown (green indicators). (a) and (b) moved along the XY plane while (c) moved along the Z direction. For (c), the movement of the particles in the Z direction causes them to exit the focal plane of the microscope.

Subsequently, experiments were conducted in which a limited quantity of particles was introduced into the working volume. The aim was to achieve micromanipulation of individual particles using a combination of electrodes that would demonstrate three-dimensional manipulation capabilities. To accomplish this, configurations depicted in Fig 10 were established, which were initially simulated and subsequently tested experimentally. The outcomes of these experimental trials are illustrated in Fig 11, showcasing the effectiveness of the proposed electrode configurations in achieving precise micromanipulation of particles.

Fig 11 shows that the selected pairs of electrodes induced movement of target particles in the XY axis (Fig 11A and 11B), and in the Z direction (Fig 11C), which both correspond to the simulations shown in Fig 10. Owing to limitations in the acquisition setup, movement in the Z direction is shown as a particle moving away from the focal plane of the microscope. The videos corresponding to these experiments compared to the simulations are available in S1–S3 Videos.

Another of the platform’s capabilities is further demonstrated in S4 Appendix and S4 Video, where we conduct proof of concept negative and positive dielectrophoresis experiments. These tests utilize a combination of particle-medium and DEP parameters such that either DEP force polarity can be selected by varying the frequency of the applied signal.

## Conclusions

This study has successfully demonstrated the design, simulation, fabrication, and testing of a novel C-MEMS platform. The platform leverages the use of carbon materials to easily produce a microelectrode array capable of achieving three-dimensional electrokinetic micromanipulation of microparticles within a microvolume, as C-MEMS technology allows for the straightforward fabrication of both 2D and 3D biocompatible microstructures. The use of a transparent substrate enhances the platform’s versatility, allowing for the integration of optical and electrokinetic techniques to control a wide range of microparticles, from individual entities to large collections.

Extensive experiments were conducted to validate the effectiveness of the proposed C-MEMS platform. These experiments, involving the manipulation of fluorescent nylon microspheres, confirmed that the platform could accurately control particle trajectories in three dimensions. The experimental results closely aligned with the simulation predictions, demonstrating the platform’s capability to manipulate microparticles precisely along the desired paths dictated by the dielectrophoretic forces generated between the microelectrodes

We have also identified some limitations of our approach, particularly for particle tracking, which is hindered for particle movements along the z-axis. Future work should focus on particle tracking accuracy. Additionally, implementing a closed-loop control scheme with real-time feedback could further refine the platform’s benefits, allowing dynamic adjustments to the microelectrode configurations to maintain precise control over particle trajectories.

The versatility of the proposed C-MEMS platform opens up numerous possibilities for its application in various fields, particularly in life sciences. Potential uses include the generation of custom tissue samples for bioengineering, precise positioning of critical cell types within scaffolds, and studying the mobility differences between healthy and diseased cells. This platform represents a significant advancement in microfluidic technologies, offering a robust tool for next-generation biomedical research and applications.

## Supporting information

S1 Appendix C-MEMS device schematics. (PDF)

S2 Appendix Electrode array parameters used in the finite element analysis and proof-of-concept test videos. (PDF)

S3 Appendix Fabrication methodology and parameters. (PDF)

S4 Appendix Proof-of-concept using negative and positive DEP micromanipulation. (PDF)

S1 Video Test configuration a. (MP4)

S2 Video Test configuration b. (MP4)

S3 Video Test configuration c. (MP4)

S4 Video Proof-of-concept using nDEP and pDEP micromanipulation. (MP4)
