# Supplementary material for: Beyond two dimensions: Exploring 3D dielectrophoresis for microparticle control using carbon electrodes
Source: PLoS One. 2024 Sep 26;19(9):e0310978. doi: 10.1371/journal.pone.0310978 (PMC11426537; doi:10.1371/journal.pone.0310978)
Supplement: S1 Appendix — (PDF) [file pone.0310978.s001.pdf]

C-MEMS device schematics

The following designs have been reproduced with permission from (Pilloni O. Micro Platform for the Manipulation of Bioparticles Using Nonuniform Electric Field Electrokinetics. 2019). All dimensions in mm.

Layer 1: Electrical traces and pads

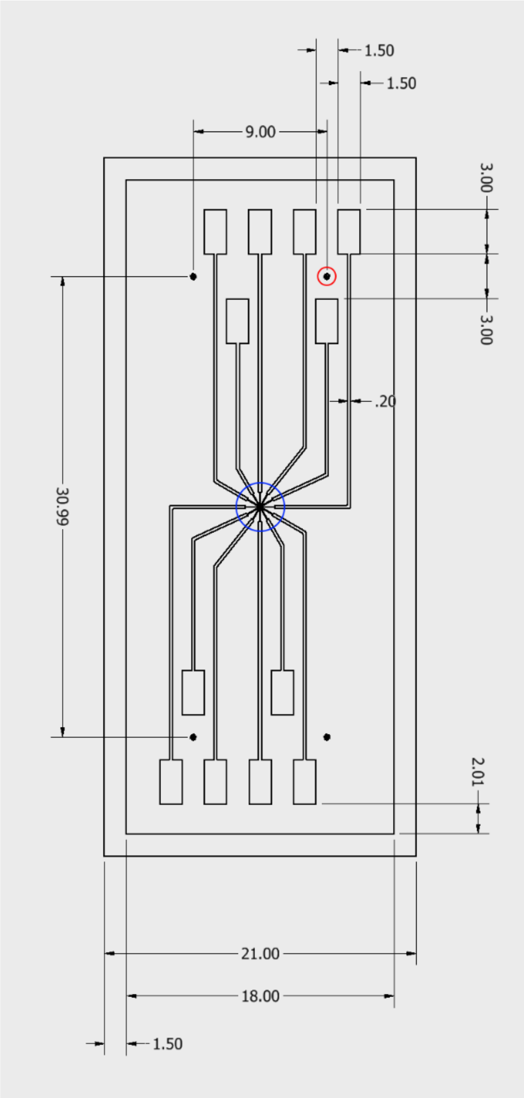

Fig 1. Layer 1.

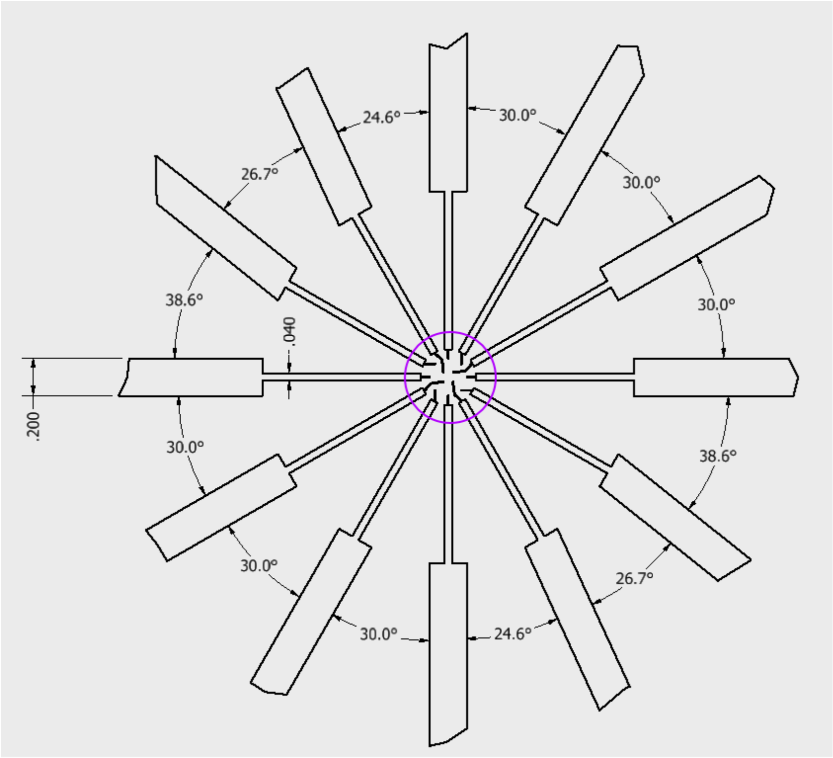

Fig 2. Blue detail from layer 1.

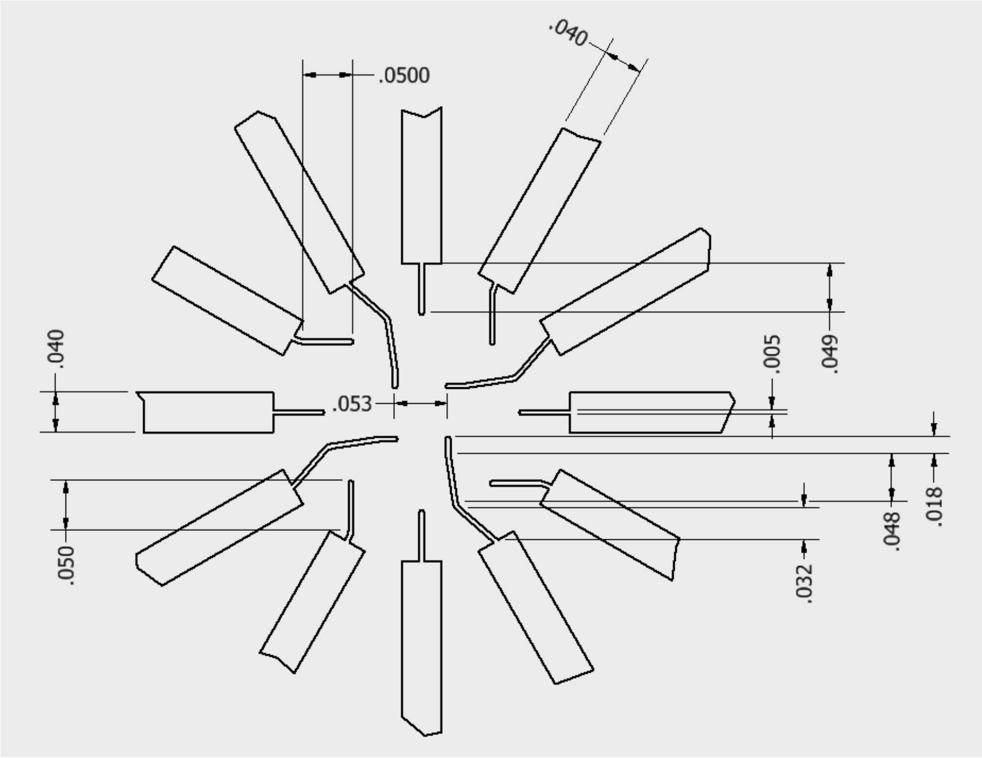

Fig 3. Purple detail from Fig 3.

Layer 2: 2D and 3D microelectrode bases

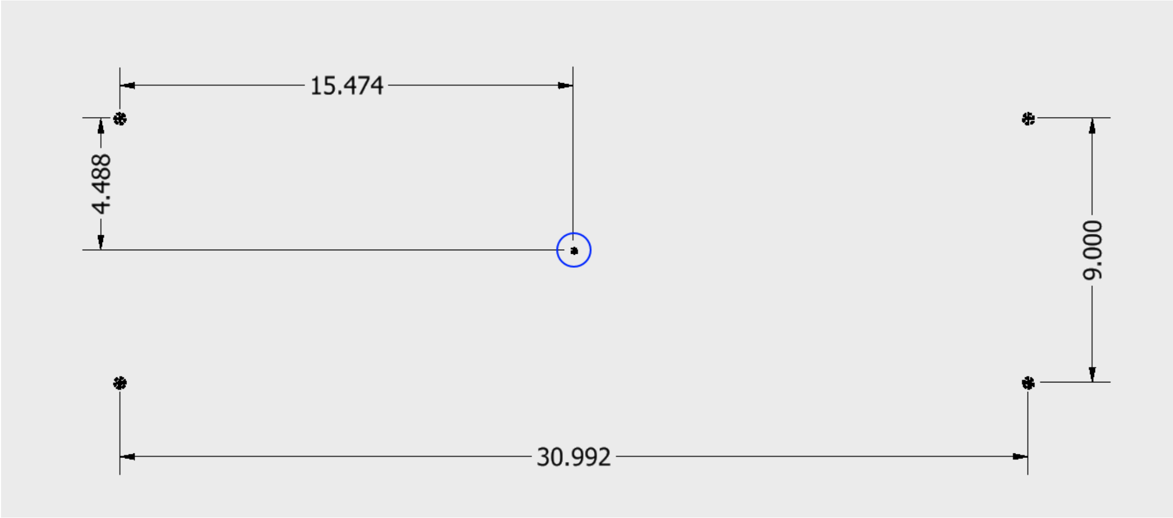

Fig 4. Layer 2.

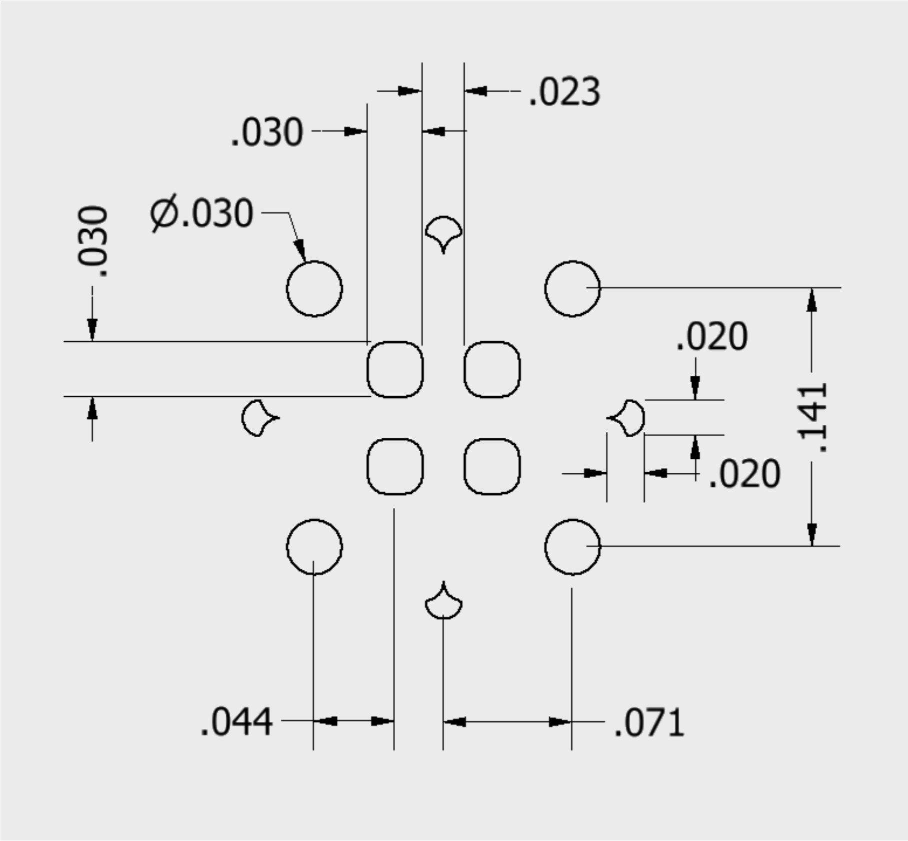

Fig 5. Blue detail from layer 2.

Layer 3: 3D microelectrodes

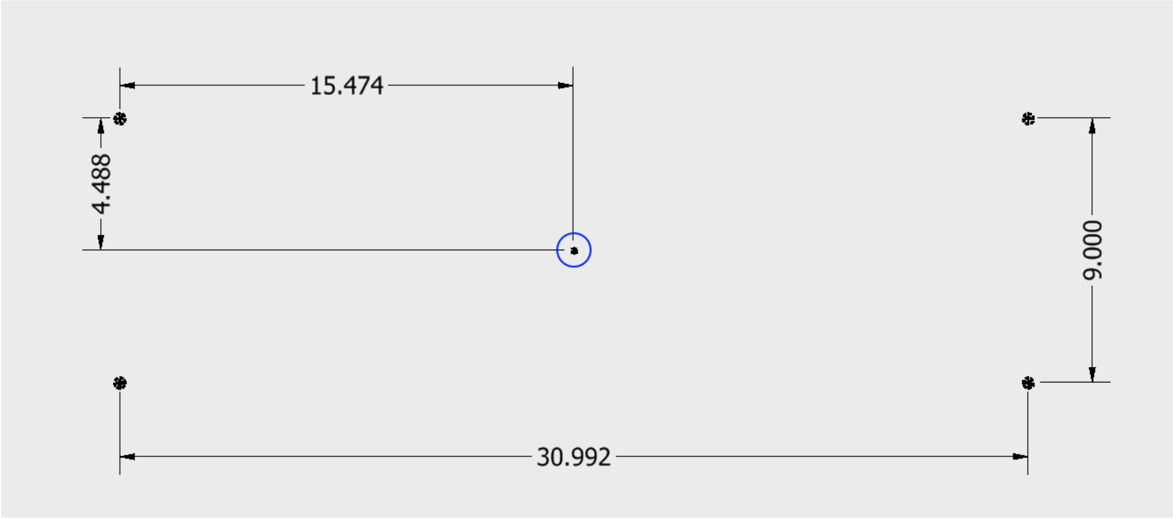

Fig 6. Layer 3.

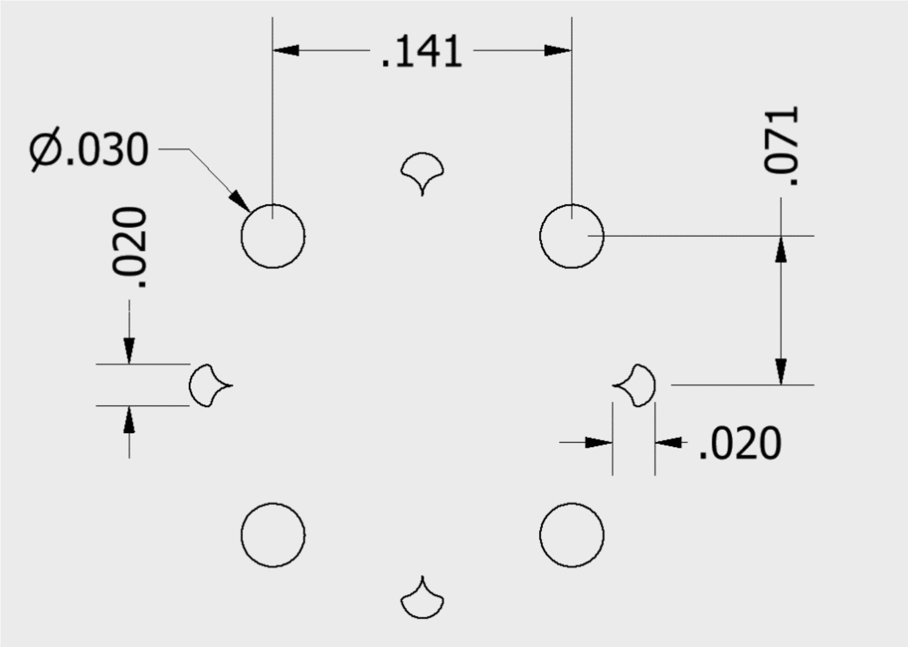

Fig 7. Blue detail from layer 3.

Layer 4: Passivation

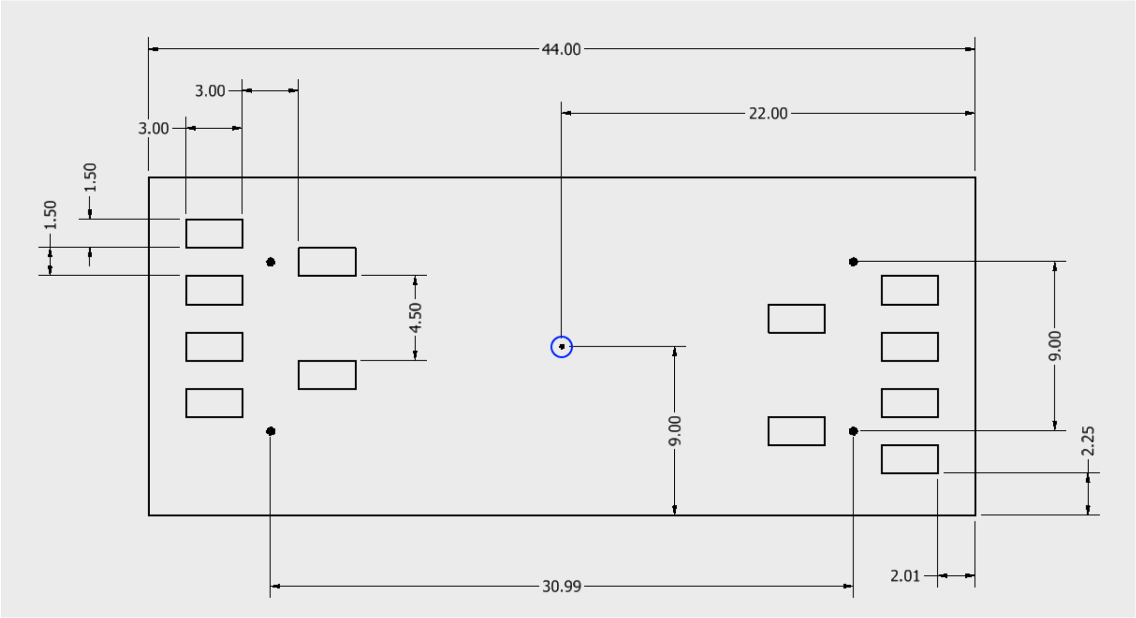

Fig 8. Layer 4.

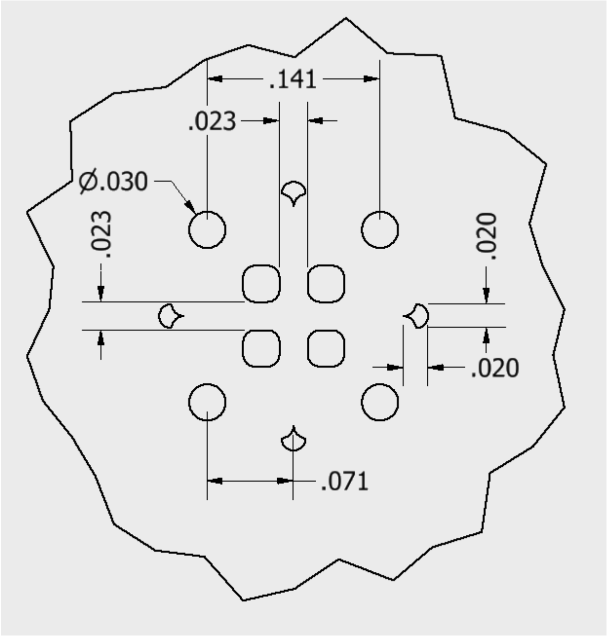

Fig 9 Blue detail of layer 4.

Layer 5: Microchannel

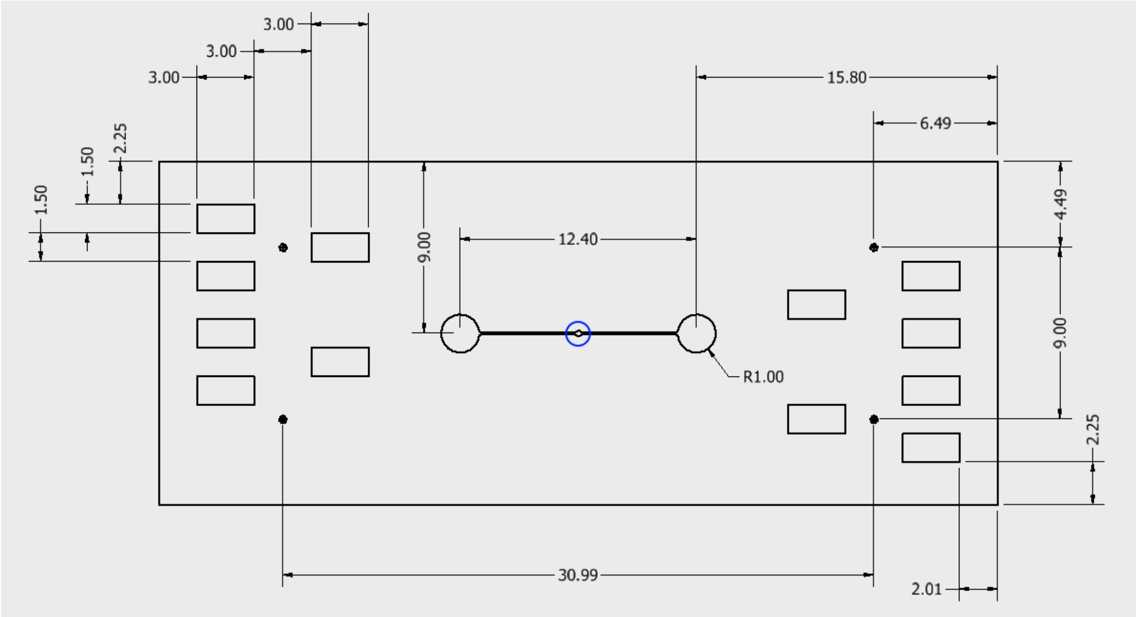

Fig 10. Layer 5.

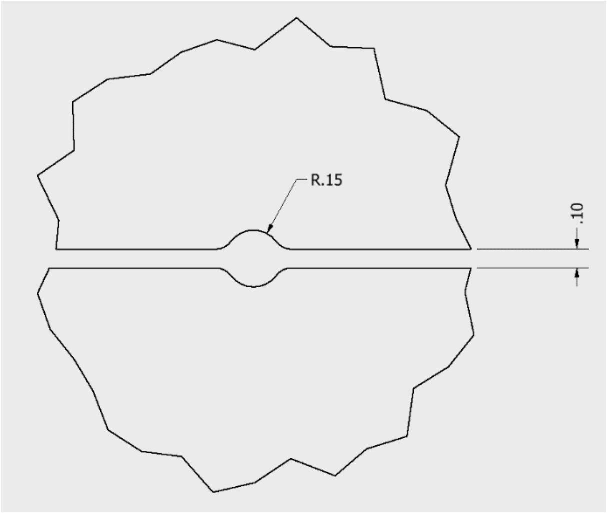

Fig 11. Blue detail from layer 5.

PDMS chip

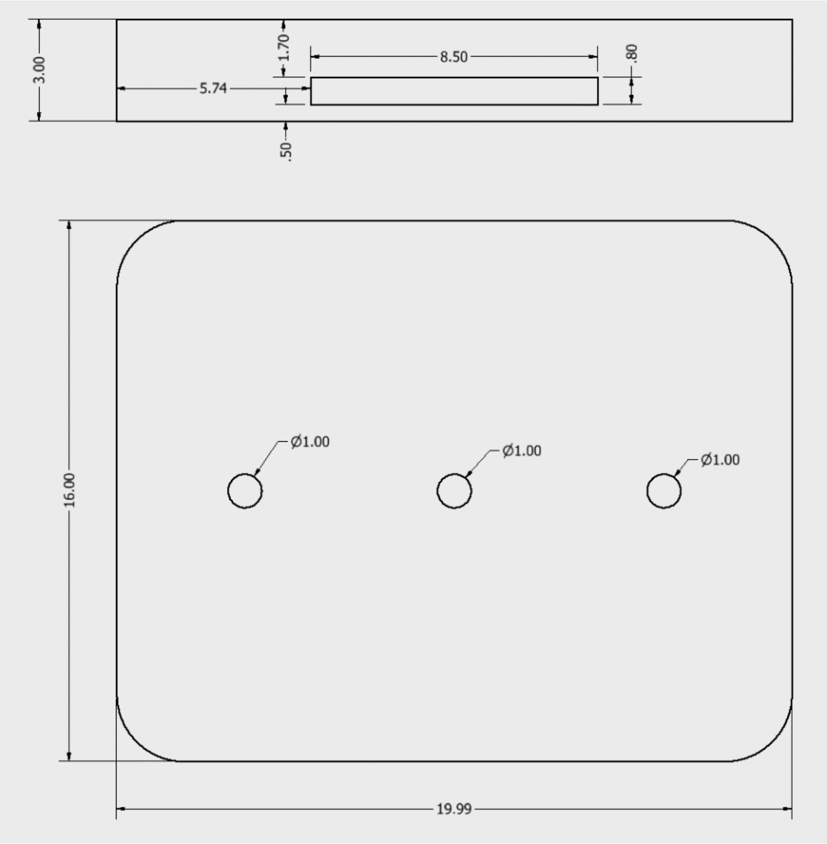

Fig 12. PDMS chip schematics.

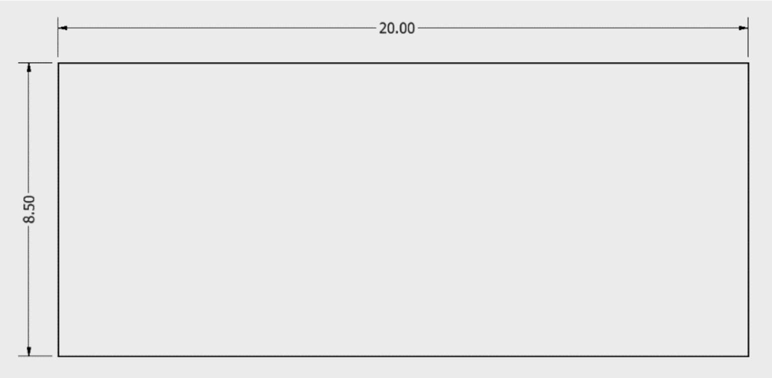

Fig 13. ITO-covered glass slide dimensions. Thickness = 0.8 mm.

PDMS chip mold

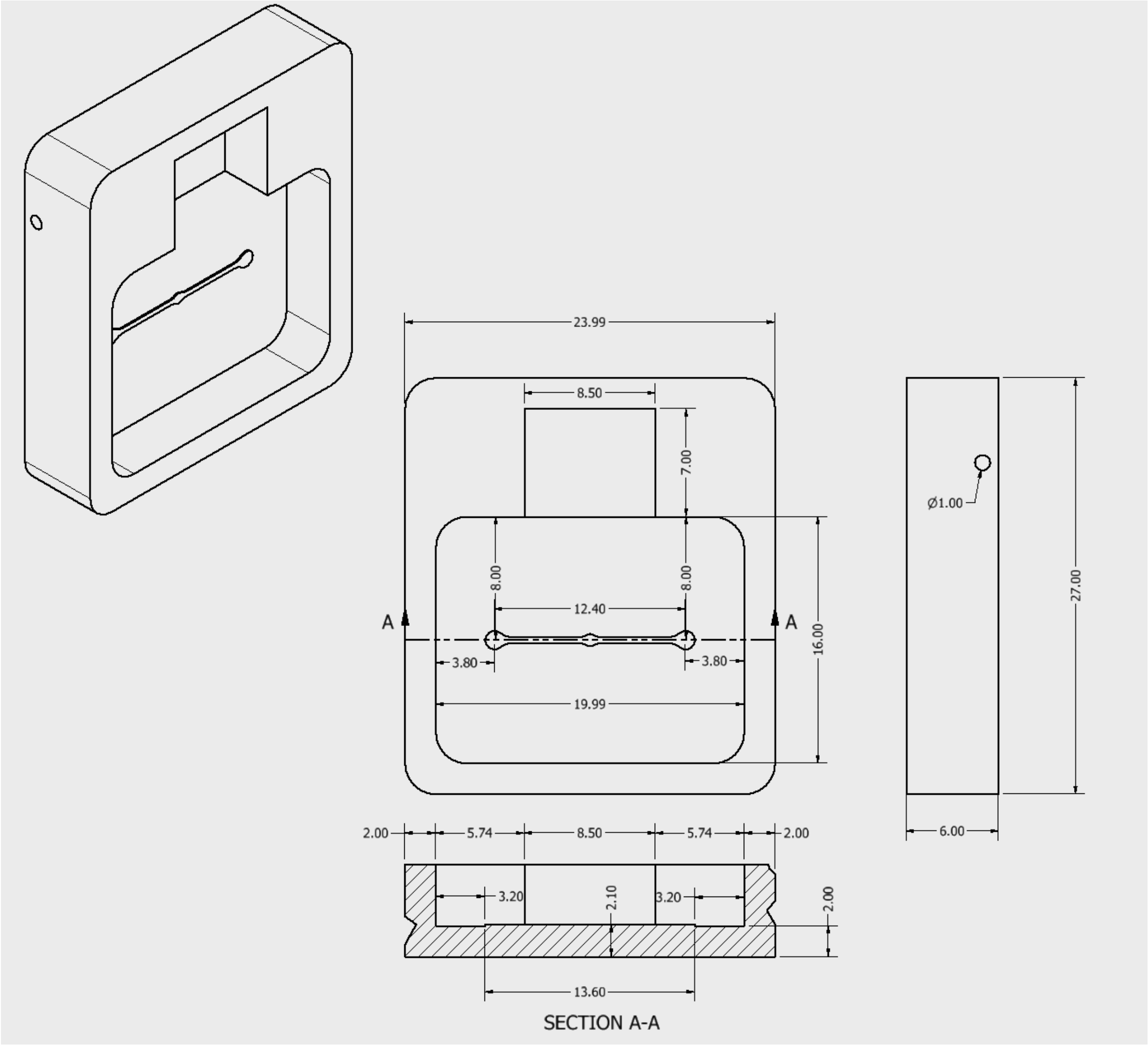

Fig 14. PDMS chip mold schematics.
