# Supplementary material for: Beyond two dimensions: Exploring 3D dielectrophoresis for microparticle control using carbon electrodes
Source: PLoS One. 2024 Sep 26;19(9):e0310978. doi: 10.1371/journal.pone.0310978 (PMC11426537; doi:10.1371/journal.pone.0310978)
Supplement: S2 Appendix — (PDF) [file pone.0310978.s002.pdf]

Electrode array parameters used in the finite element analysis and proof-of-concept test videos

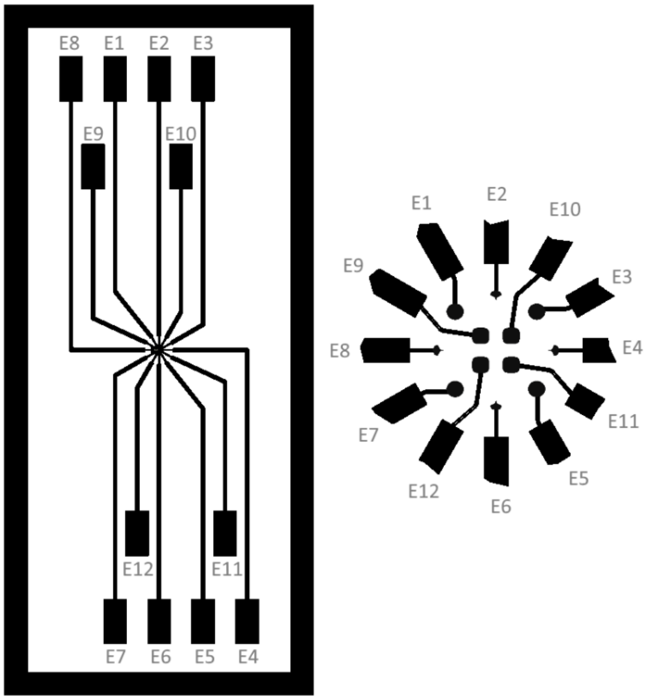

Fig 1. Pinout and designator names for each microelectrode. Top counter electrode, made of an ITO coated slide is referred to as top C.E. (not shown).

Table 1. Electrode array configurations used in the experiments.

Array configuration	V_{DEP} Electrodes	GND Electrodes
a	E1	E5
b	E7	E10
c	E12	Top C.E.

The videos S4 Video a, S5 Video b and S6 Video c correspond to test cases (a), (b) and (c). V_{DEP} Electrodes were electrically connected to a signal generator (15 V, sine wave, 100kHz). GND Electrodes were connected to electrical ground. Other unused electrodes were tied to a high impedance lane. Electrode designator names are used according to Fig 1.
