# Supplementary material for: Beyond two dimensions: Exploring 3D dielectrophoresis for microparticle control using carbon electrodes
Source: PLoS One. 2024 Sep 26;19(9):e0310978. doi: 10.1371/journal.pone.0310978 (PMC11426537; doi:10.1371/journal.pone.0310978)
Supplement: S3 Appendix — (PDF) [file pone.0310978.s003.pdf]

Fabrication methodology and parameters

Multilayer photolithography was employed to pattern the microelectrode array. The device fabrication process required five layers. Layers 1-3 formed both planar and volumetric microelectrodes. These layers were then pyrolyzed. Layer 4 forms a passivation layer (Fig 1), and layer 5 forms a microfluidic channel surrounding the array.

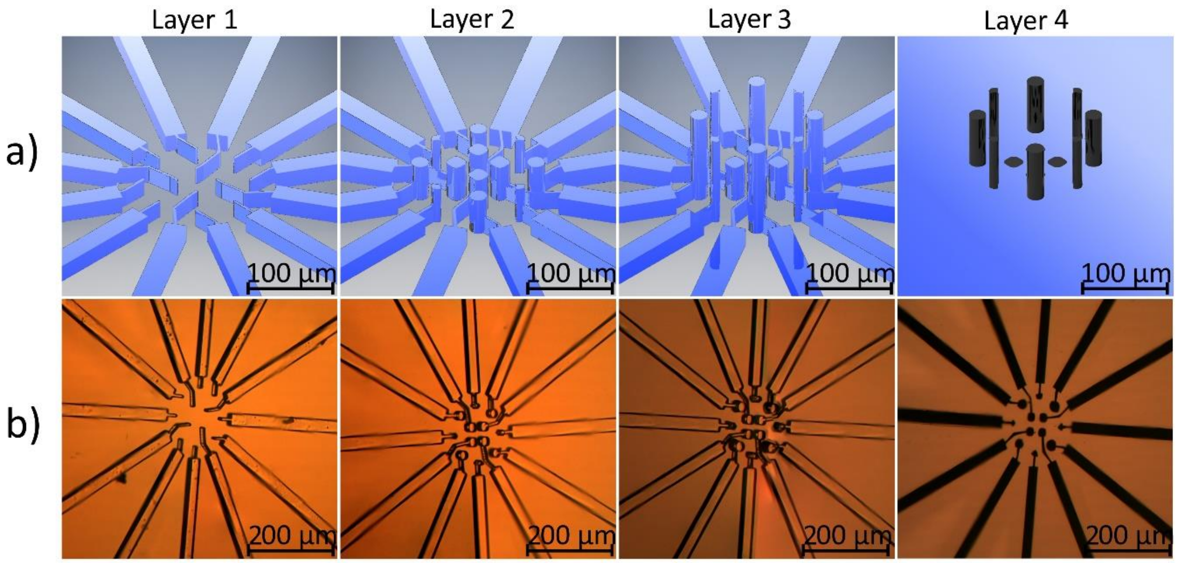

Fig 1. Sequential (a) illustration and (b) photography guide to microelectrode array fabrication. The process begins with patterning Layers 1-3 using SU-8 photoresist (blue/translucent) to form the base of the microelectrode array. Following pyrolysis, which transforms these layers into carbon microstructures (Layer 4), an additional SU-8 layer is applied to create a protective passivation layer. Lastly, Layer 5, not shown, is deposited to construct a surrounding microchannel for isolation and protection.

Substrate Cleaning

To clean the substrate (Fused Silica, JGS2 grade, 500 μm thick, University Wafer Inc), a layer of acetone was sprayed on the surface of the substrate and then a spin coater (WS-650Mz-23NPP, Laurell) was used to spin it at 2000 RPM. This process was repeated using isopropyl alcohol. The substrate was then placed on a hot plate at 230 °C for one hour to dehydrate it. Afterwards, the substrate was removed from the hot plate and was allowed to cool to room temperature (18 °C).

Photoresist deposition

The substrate was spin-coated with SU-8 3035 (Microchem) using a two-step spin velocity program. The time and velocity were selected to produce the film thickness required for each designed layer (Table 1).

Table 1. Spin coater parameters for photoresist deposition.

**Layer**	**Thickness [μm]**	**Step 1**	**Step 2**
1	35	15s @ 500 RPM	45s @ 4000 RPM
2	35	15s @ 500 RPM	45s @ 4000 RPM
3	100	15s @ 500 RPM	45s @ 1000 RPM
4	40	15s @ 500 RPM	45s @ 3000 RPM
5	100	15s @ 500 RPM	45s @ 1000 RPM

Soft bake

The substrate was placed on a hot plate (HP131534, Thermo Scientific), preheated to 65 °C, and then heated at a rate of 10 °C/min to 95 °C. The substrate was then left to bake at this temperature for 30 min. (35 and 40 µm layers) or 60 min (100 µm layers), depending on the thickness of the deposited film. The hot plate temperature was then decreased at a rate of 10 °C/min until the room temperature was reached.

Exposure

A maskless exposure machine (SF-100 XCEL, Intelligent Micro Patterning) is used to expose the desired pattern. It was configured to have a wavelength of 365 nm. The exposure lens, time, and offset per layer are presented in Table 2.

Table 2. Layer exposure parameters for the mask-less exposure machine.

Layer	Lens	Time [s]	Offset [mm]
1	4x	7.1	+0.043
2	20x	0.71	-0.001
3	20x	0.8	-0.001
4	4x	7.1	+0.043
5	4x	7.1	+0.043

Post exposure bake

The substrate was then placed on a hot plate and preheated to 65 °C. The temperature was increased to 95 °C at a rate of 10 °C/min. The substrate was then maintained at this temperature for 5 min. (35 and 40 µm layers) or 10 min (100 µm layers), depending on the thickness of the deposited film. Subsequently, the substrate was cooled at a rate of 10 °C until room temperature was reached.

Development

The substrate was immersed in a crystallizer with sufficient developer (Microposit SU8 Developer) to completely cover its surface. It was subsequently subjected to moderate agitation by hand for 3-8 minutes depending on the layer thickness. After this, the substrate was removed from the developer solution and rinsed with IPA, followed by rinsing with fresh developer solution. Subsequently, the substrate was rinsed again with IPA and dried using nitrogen (99.9999% pure).

Hard bake

The substrate was gradually heated on a hot plate at a rate of 10 °C/min from room temperature to 190 °C and held constant for one hour. The temperature was then decreased at the same rate until it reached room temperature. This process was performed only after the deposition of layers 3 and 5.

Pyrolysis

Pyrolysis was performed only after the deposition of layer 3. It was placed in a quartz tube inside a tube furnace (OTF-1200X-80-SL, MTI Corporation) under vacuum.

First, the substrate was placed inside the reaction chamber, and air was pumped out of the chamber until a vacuum of 4 mTorr was reached. This vacuum was maintained for 20 min before the furnace was turned on. The furnace was subsequently heated according to the heating profile shown in Fig 2. Finally, the furnace was turned off after the heating profile was completed, and the substrate was left inside to cool naturally to ambient temperature before extraction.

Fig. 3 illustrates the photolithography and pyrolysis processes.

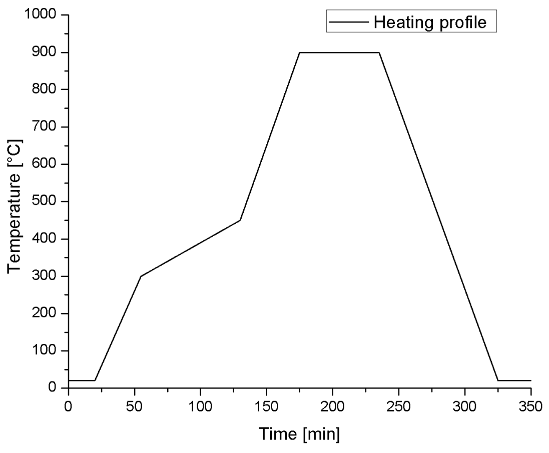

Fig 2. Heating profile for the pyrolysis process. This process was only performed after layer 3 was deposited to form the microelectrode array.

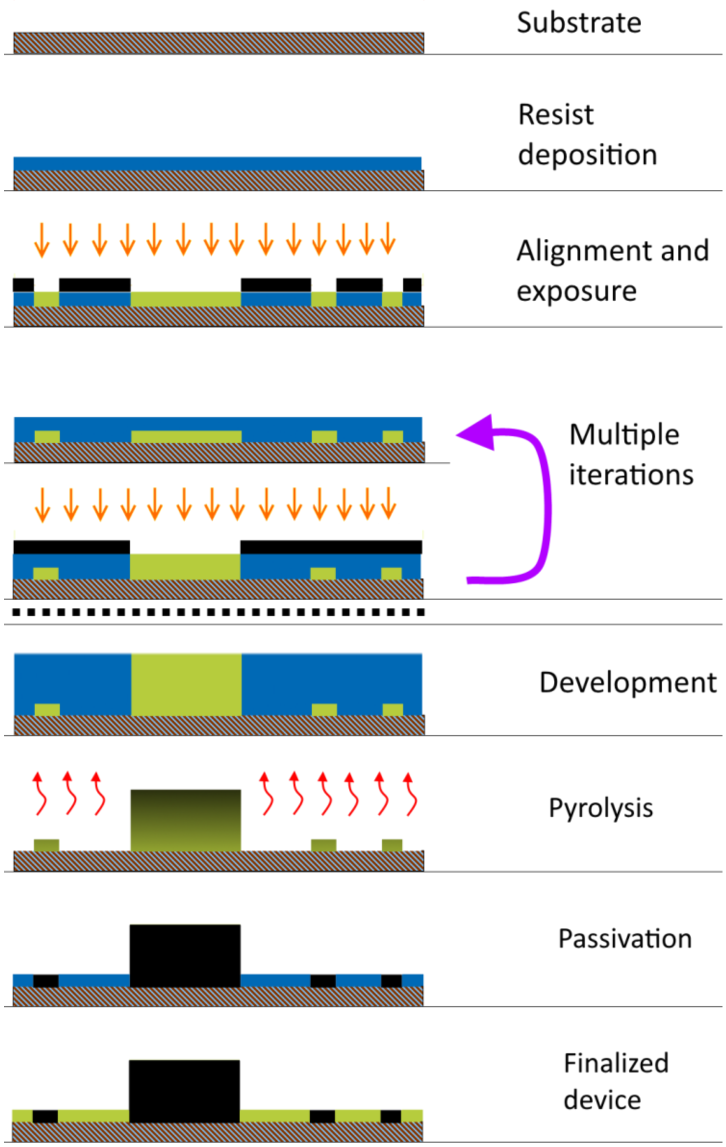

Fig 3. Photolithography and pyrolysis fabrication steps. Layers 1-3 were fabricated through successive photoresist deposition and exposure processes. Afterward, the unexposed photoresist was developed, and the remaining structures were pyrolyzed to form the carbon features. Subsequently, layers 4 and 5 were applied to passivate the conductive carbon traces, thereby completing the device fabrication.
