# Supplementary material for: Beyond two dimensions: Exploring 3D dielectrophoresis for microparticle control using carbon electrodes
Source: PLoS One. 2024 Sep 26;19(9):e0310978. doi: 10.1371/journal.pone.0310978 (PMC11426537; doi:10.1371/journal.pone.0310978)
Supplement: S4 Appendix — (PDF) [file pone.0310978.s004.pdf]

Proof-of-concept using negative and positive DEP micromanipulation

The micro platform was mounted in a TCM 400 fluorescence inverted microscope for observation and then connected to the control electronics module. A CMOS camera (M2, Pixelink) was used to record the experiment. Electrical parameters of the DEP signal were varied to exert either positive (pDEP) or negative (nDEP) dielectrophoresis on a target particle. For the particle-medium pair, 1 µm polystyrene fluorescent microparticles (FluoSpheres) were suspended in deionized water. Their properties are summarized in Table 1.

Table 1. Suspension media and microparticle electrical properties.

**Properties**	**Value**
**Suspension media**	**Deionized and distilled water**
Electrical conductivity	$\sigma_m = 2x10^{-8}\frac{S}{m}$
Relative electrical permittivity	$\epsilon_m = 80$
**Microparticles**	**FluoSpheres (polystyrene)**
Diameter	$\phi = 1\ \mu m$
Electrical conductivity	$\sigma_p = 8x10^{-4}\frac{S}{m}$
Relative electrical permittivity	$\epsilon_p = 2.56$

Properties used to calculate the Clausius-Mossotti factor (K). This provided the necessary information for the selection of suitable frequencies for both pDEP and nDEP.

The following electrical signal characteristics were used for this experiment. pDEP and nDEP frequencies were selected using Fig 1.

- Electric signal applied
  - Electric potential $V_{DEP} = 10\ V$
  - Signal source = external
  - Signal type = sine
  - pDEP Signal frequency $f = 10\ kHz$
  - nDEP Signal frequency $f = 500\ kHz$

During the experimentation process, the dielectrophoretic force was alternated between positive and negative, selecting the appropriate frequency. This shift effectively attracted or repelled the microparticles from the active electrodes. Visualization of this dynamic process can be found in S8 Video pDEP.

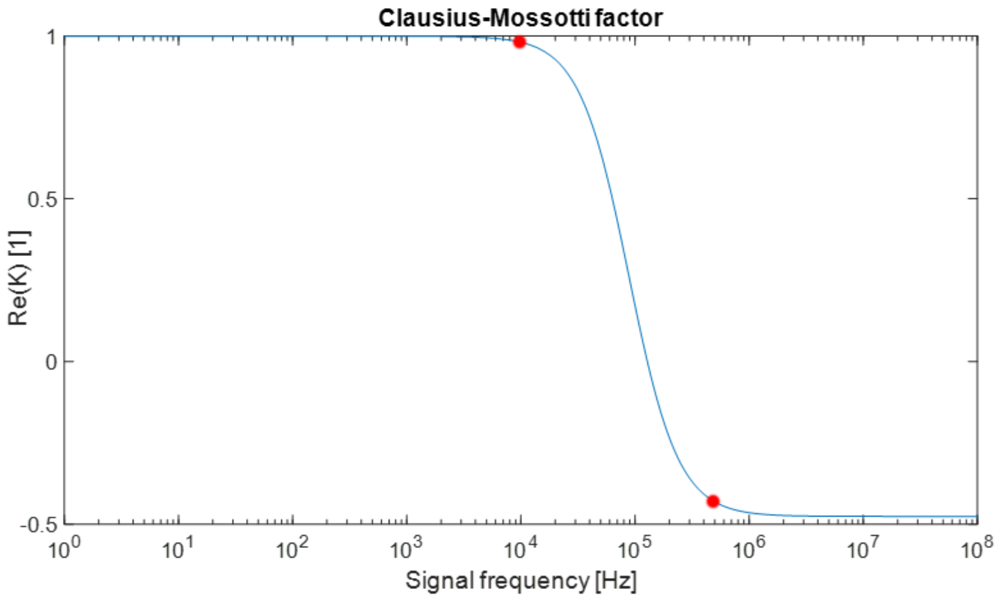

Fig 1. Real part of the Clausius-Mossotti factor (K) for a wide range of frequencies. Re[K] was calculated across a broad frequency spectrum to aid in the selection of signal frequencies capable of exerting both types of dielectrophoretic (DEP) forces. From this calculation, 10 kHz was specifically chosen for positive dielectrophoresis (pDEP), while 500 kHz was selected for negative dielectrophoresis (nDEP). Chosen frequencies are highlighted with red dots.
